# Modeling of formation damage during smart water flooding in sandstone reservoirs

**DOI:** 10.1038/s41598-023-44160-0

**Published:** 2023-10-16

**Authors:** Mohammad Amin Bagrezaie, Bahram Dabir, Fariborz Rashidi, Ali Reza Moazzeni

**Affiliations:** 1 Department of Petroleum Engineering, Amirkabir University of Technology, Hafez Street, PO Box 158754413, Tehran, Iran; 2 Department of Chemical Engineering, Amirkabir University of Technology, Hafez Street, PO Box 158754413, Tehran, Iran

**Keywords:** Chemical engineering, Computational science, Crude oil, Software

## Abstract

Impairment of permeability has been observed as an effective factor in production decline during secondary and tertiary recovery processes such as water flooding. Among different permeability damage mechanisms, fines migration and deposition is known as the main mechanism which occurs due to pore throat clogging and blocking. Because injected water and formation water are usually incompatible, permeability damage evaluation and scale formation prediction must be done before the water flooding process in the oil field is implemented. For this purpose, compatibility tests and core flood experiments are common, but experimental approaches with time and facility limitations are expensive. Thus, by decreasing the time required for conducting experiments, modeling approaches can replace the routine laboratory experiments. Based on thermodynamic balance and the solubility of ions in water, scale development due to seawater injection in an Iranian oil field was predicted in this work using the OLI ScaleChem software. After that, it was suggested that special water be introduced to help reduce the amount of scales that had accumulated in the rock pore space. The extent of permeability damage in various seawater injection scenarios was then assessed via dynamic core flood experiments. Finally, scales-seawater injection into the core was simulated using digital core analysis (DCA) results and the pore scale modeling approach. The core flood experiment data are consistent with the scale formation prediction made by the OLI ScaleChem software, which indicates that smart water can be determined by optimizing the salinity and ion content of injected water. Also, results of permeability damage prediction by our modeling approach have good agreement with the core flood experiment data. Therefore, our modeling approach can replace the conventional core flood experiments as a low-cost method with high computational efficiency and high enough accuracy to evaluate formation damage in the water flooding process.

## Introduction

Due to the availability of seawater and the fact that it can be used in either the early or late stages of field development, water flooding is one of the most popular IOR techniques in offshore fields^1–3^. Implementing this process depends on the extent of formation damage due to mineral scaling, which can lead to the technical failure of this method. Scale formation in the reservoir rock and precipitation of these scales leads to clogging and blocking of the pore throat and eventually permeability damage. In addition, it also leads to destruction of equipment in the production system. Therefore, to assess and manage the deposition of mineral scale, a study of the incompatibility between injected water and formation water is required. Ionic composition, salinity, pressure, temperature, and pH are the primary parameters in the scale deposition analysis^4,5^.

**Figure 1:**
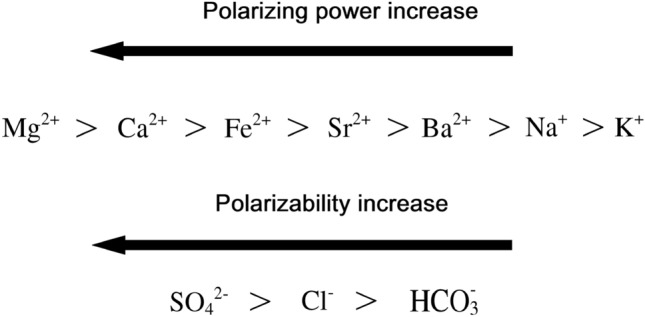
Polarizing power of cations vs polarizability of anions.

Smart water selection refers to a recent study aimed at techniques to prevent or limit the formation of scale by altering the ion composition and salinity of injected water^6,7^. Generally, sea and formation waters may contain anions such as sulfate (SO_4_^−2^), chloride (Cl^−^), and bicarbonate (HCO_3_^−^) and cations such as magnesium (Mg^+2^), calcium (Ca^+2^), ferrous (Fe^+2^), strontium (Sr^+2^), barium (Ba^+2^), sodium (Na^+^), and potassium (K^+^). Therefore, based on ionic concentration and ionic potential, various scales may form. In fact, ionic concentration controls the amount of scale and ionic potential controls the type of scale. Ionic potential refers to power of cation to polarize the anion and form an ionic network (scale) by ionic bond. When cation with a higher positive charge and smaller size polarizes the anion with higher negative charge and larger size, then the ionic potential is maximal. Hence, among the various possible scales, a scale will be formed first in which the ionic potential between the cation and the anion is maximal. The polarizing power of different cations as well as the polarizability of different anions is given in Fig. 1.

Therefore, in the same condition based on ionic potential, the possibility of sulfate scale formation is more than chloride and carbonate scales. However, precipitation of chloride scales because of solubility in water is not a concern. Thus, the high concentration of sulfate (SO_4_^−2^) anion in seawater and divalent cations in the formation water led to precipitation of harmful scales except for magnesium sulfate (MgSO_4_), which is soluble in water and will not damage the formation. In fact, the high charge density of magnesium cation led to high hydration energy and the great difference in ionic size between magnesium cation and sulfate anion led to low lattice enthalpy, resulting in the very low solubility of magnesium sulfate (MgSO_4_) in water. Moreover, the low concentration of sulfate (SO_4_^−2^) anion in comparison to bicarbonate (HCO_3_^−^) anion led to precipitation of carbonate scales. Calcium sulfate (CaSO_4_), strontium sulfate (SrSO_4_), barium sulfate (BaSO_4_), calcium carbonate (CaCO_3_), and ferrous carbonate (FeCO_3_) are the most prevalent scales in Iranian offshore oil fields. These substances can precipitate as both large and minor scales at the same time^8–13^. The deposition probability of different mineral scales on the reservoir rock surface and consequent permeability damage has been studied by many researchers^8–10,14,15^.

Nowadays, digital core analysis can replace the expensive core flooding experiments as a rapid and cheap method and also provide a better understanding of pore scale properties of reservoir rock, especially in the sandstone core due to lower heterogeneity. This method uses data from scanning electron microscopy (SEM), thin-section micrographs, plug micro-CT, and whole-core computed tomography (CT) to estimate reservoir rock attributes, including porosity, grain size distribution, pore/throat size distribution, and others. Then the pore network modeling approach will be considered for pore scale simulations of flow in the core^16–18^.

**Figure 2:**
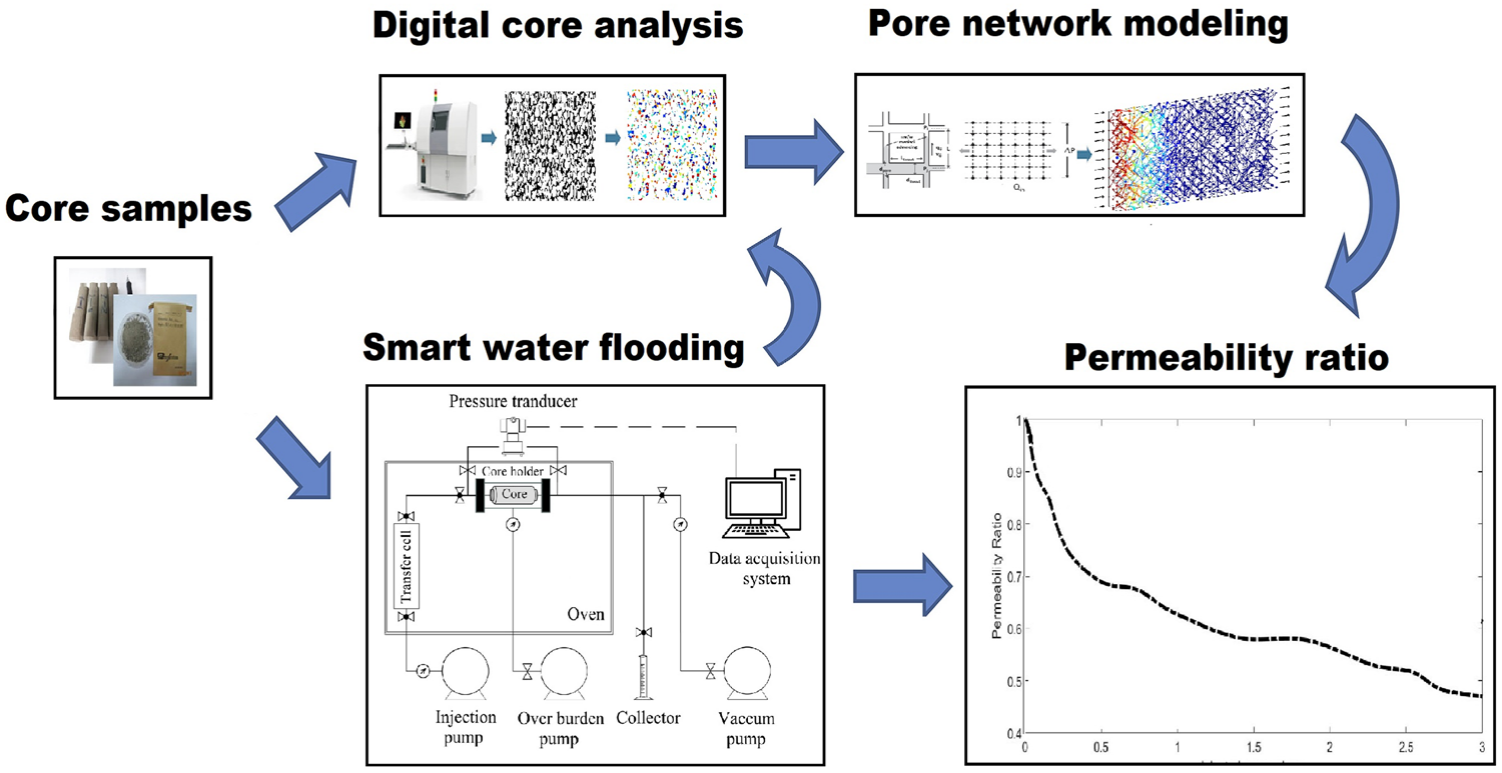
Research workflow.

In this study, water flooding was evaluated in an Iranian oil reservoir. For this purpose, the amount of seawater salinity and seawater composition was initially optimized by OLI ScaleChem software which considers the thermodynamic aspects of mineral scale formation. Then, the amount of permeability damage due to seawater injection into the core was investigated by both modeling and experimental approaches. A 2-D pore network model based on porosity, pore size distribution, and throat size distribution was created using the modeling approach as a random distribution in accordance with the core dimension. A dual pore scale model was created using the MATLAB software to calculate pressure drop and the permeability damage in the pore network model. Additionally, the amount of permeability damage caused by the optimum seawater (smart water) injection and seawater injection were determined and compared using experimental approaches. The mechanism of smart water flooding in oil reservoirs is very complex due to different effective parameters such as formation mineralogy, formation water salinity and composition, and reservoir temperature. Therefore, our model is only applicable to the target reservoir in this study, but our approach can apply to all oil reservoirs. In short, our approach includes modeling of particle-fluid flow in pore space using the modified DPM model, proxy modeling based on the ANN method, and modeling of particle-fluid flow in porous media using the PNM method alongside with scales formation evaluation and digital core analysis. Figure 2 shows the workflow of this research. Our modeling methodology can be used as an alternate way for core flooding experiments when there are time and facility constraints based on a good match between the modeling approach results and core flooding data.

## Methodology

### Smart water determination

The OLI ScaleChem software was used in this study to gain the smart water by determining its optimum salinity and composition. This software can forecast the amount of precipitated scales and the potential for mineral scaling under both pressure and temperature conditions. In order to forecast mineral scaling during water injection, the thermodynamic balance and ion solubility in water were actually investigated.

#### Optimum salinity of injected water

**Figure 3:**
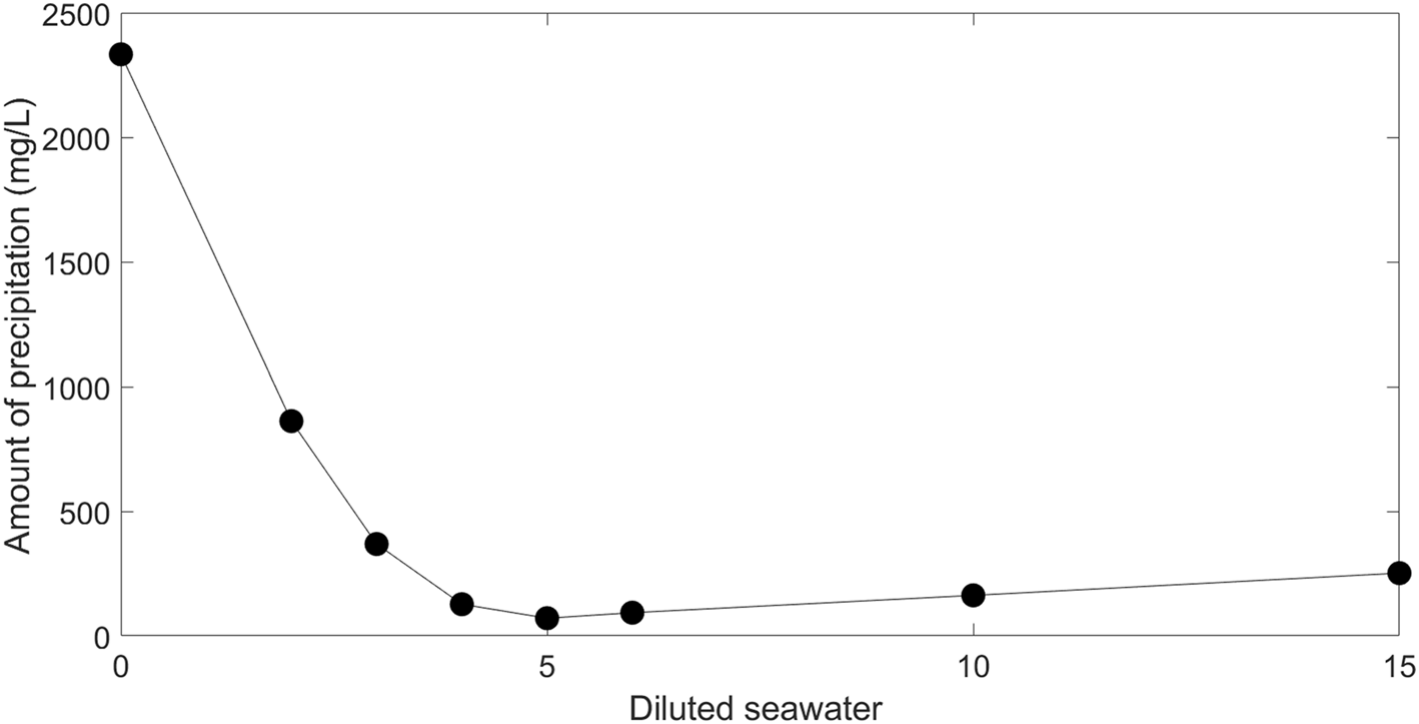
Precipitation amount of diluted seawater.

Equal mixtures of formation water and diluted seawater were taken into consideration in six situations of 2, 4, 5, 6, 10, and 15-times dilution in order to determine the optimum salinity of the injected water. Then, using OLI ScaleChem software, the amount of precipitation in each combination was assessed. The make-up of seawater and diluted seawaters used to determine the optimum salinity is shown in Table 1. The situation with the least amount of precipitation has the best salinity of injected water. The amount of precipitation for each scenario is shown in Fig. 3 under reservoir conditions, with the best case occurring at a 5 times dilution of seawater.

**Table 1:** Composition of different diluted seawater.

Composition	Seawater (mg/L)	2 × diluted seawater (mg/L)	4 × diluted seawater (mg/L)	5 × diluted seawater (mg/L)	6 × diluted seawater (mg/L)	10 × diluted seawater (mg/L)	15 × diluted seawater (mg/L)
Na^+^	16,814.54	8407.27	4203.63	3362.9	2802.42	1681.45	1120.96
Ca^2+^	596.5	298.25	149.12	119.3	99.41	59.65	39.76
Mg^2+^	1945	972.5	486.25	389	324.16	194.5	129.66
Cl^−^	29,334	14,667	7333.5	5866.8	4889	2933.4	1955.6
SO_4_^2−^	4360	2180	1090	872	726.66	436	290.66
HCO_3_^−^	184.5	92.25	46.12	36.9	30.75	18.45	12.3
TDS	53,235	26,617	13,308.75	10,647	8872.5	5323.5	3549

#### Optimum composition of injected water

**Figure 4:**
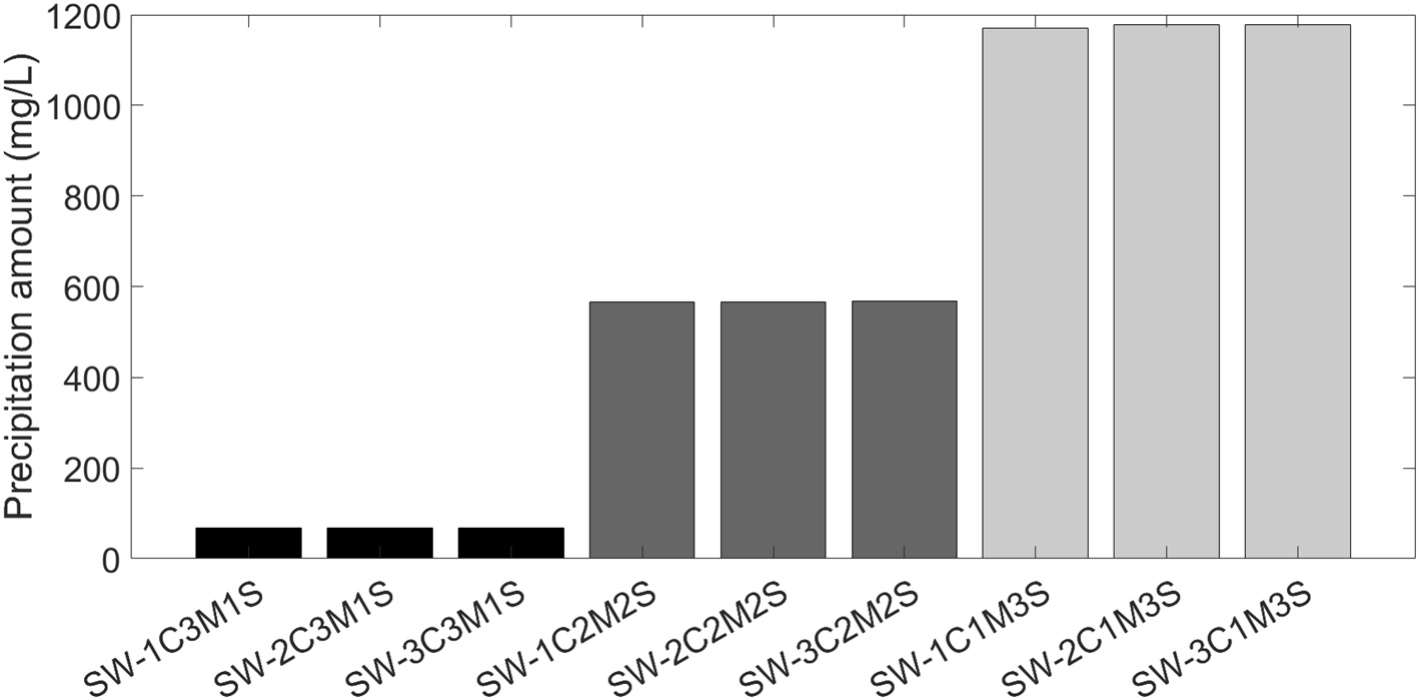
Scales precipitation amount for injected waters with different ion composition.

This section examined mineral scaling under reservoir conditions of 85 °C and 3222 psi by combining formation water and seawater at a 5 times dilution, which is the optimum salinity of injected water. Overall, nine cases with different chemical compositions for finding the optimum ion composition of injected water were considered. In each case, the concentration of Ca^2+^, Mg^2+^, and SO_4_^2−^ changed between 1 and 3 times that of seawater at 5 times dilution. For example, in the case of SW5D-1C3M1S, the concentrations of Ca^2+^, Mg^2+^, and SO_4_^2−^ of the injected water were considered as 1, 3 and 1 times those of the SW5D case. Also, the optimum ion composition of injected water occurs in a case with the lowest amount of precipitation. Table 2 shows different cases with various ion compositions. Figure 4 shows the amount of precipitation for different cases mentioned in Table 2.

**Table 2:** Chemical composition of injected water with different ion compositions.

Composition	Na^+^ (mg/L)	Ca^2+^ (mg/L)	Mg^2+^ (mg/L)	Cl^−^ (mg/L)	SO_4_^2−^ (mg/L)	HCO_3_^−^ (mg/L)	pH (mg/L)	TDS (mg/L)
SW5D	3362.92	119.3	389	5866.82	872	36.9	7.77	10,647
SW5D-1C3M1S	2163.97	119.3	1167	6287.6	872	36.9	7.65	10,647
SW5D-2C3M1S	2034.2	238.6	1167	6298.55	872	36.9	7.63	10,647
SW5D-3C3M1S	1904.1	357.9	1167	6309	872	36.9	7.62	10,647
SW5D-1C2M2S	2673.7	119.3	778	5295.18	1744	36.9	7.71	10,647
SW5D-2C2M2S	2543.6	238.6	778	5305.6	1744	36.9	7.69	10,647
SW5D-3C2M2S	2413.8	357.9	778	5316.5	1744	36.9	7.67	10,647
SW5D-1C1M3S	3183.1	119.3	389	4302.25	2616	36.9	7.79	10,647
SW5D-2C1M3S	3053.3	238.6	389	4313.15	2616	36.9	7.76	10,647
SW5D-3C1M3S	2923.21	357.9	389	4323.59	2616	36.9	7.74	10,647

**Figure 5:**
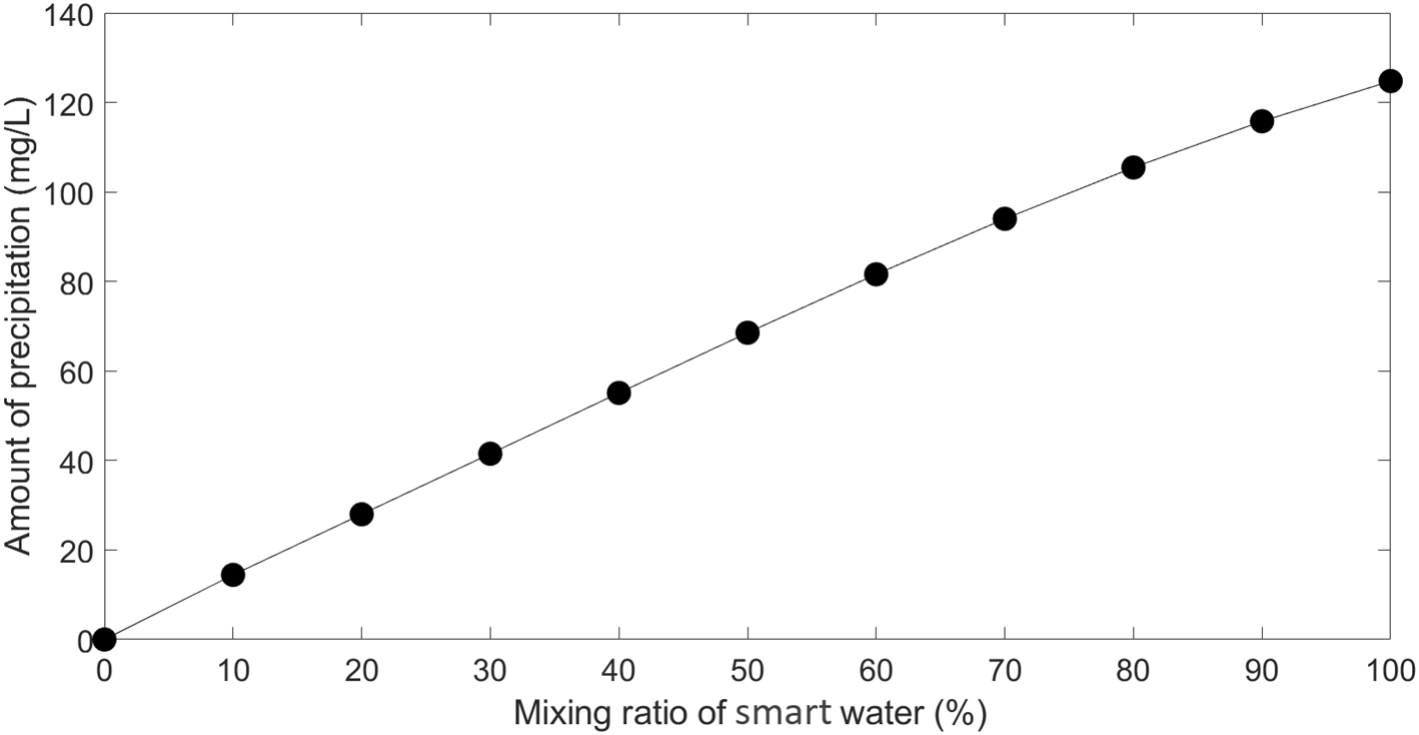
The amount of scale with ratio of seawater at reservoir condition.

Based on Fig. 4, in cases with higher concentration of Mg^2+^, the amount of precipitated scales is lowest and in cases with higher concentration of SO_4_^2−^, the amount of precipitated scales is highest. The formation of magnesium sulfate, which is soluble in water and will not damage the formation, is really caused by the magnesium ion initially creating an ion pairing with the sulfate ion because of the higher ionic potential. Therefore, a higher magnesium ion concentration results in a decrease in the quantity of sulfate ions and prevents the formation of calcium sulfate, which is the main cause of formation damage. When the sulfate ion concentration is low, on the other hand, the magnesium ion likes to form ion pairings with the carbonate ion. Because calcium carbonate is another damaging scale, a larger concentration of magnesium ions hinders its development. According to this study, a rise in the concentration of magnesium ions or a drop in the concentration of sulfate ions can help determine the smart water. SW5D-1C3M1S is the most likely smart water instance, according to software projections. Figure 5 displays the quantity of scale precipitation for the injected smart water; in this instance, only CaCO_3_ was produced, whereas Fig. 3 previously reported that the injected seawater produced CaSO_4_, CaCO_3_, and SrSO_4_ with a total precipitation amount of 2333.13 mg/L.

### Experimental study

#### Glass microchannel flooding tests

**Figure 6:**
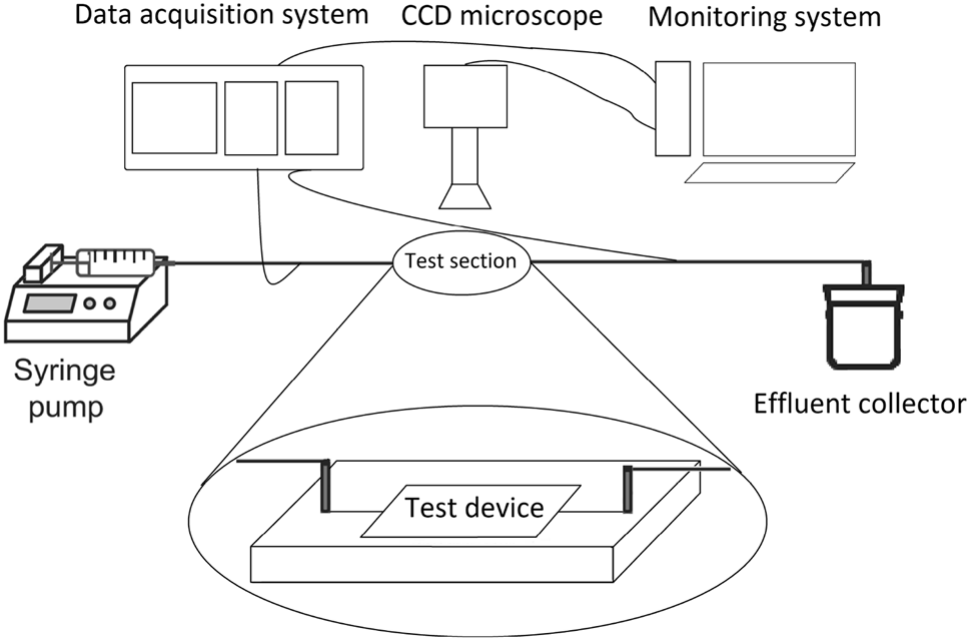
Schematic of suspension injection experimental setup (Bagrezaie et al.^24^).

**Figure 7:**
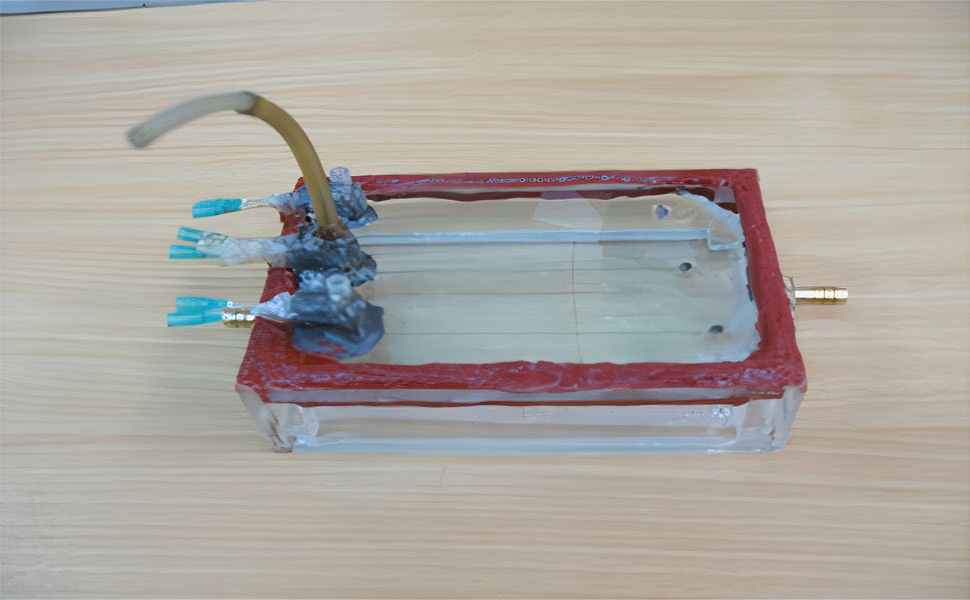
Glass test device (Bagrezaie et al.^24^).

In this work, a set of water-particle suspension injection into the glass test device was performed for modified DPM model validation. Figure 6 shows a schematic of the suspension injection experimental setup and Fig. 7 shows the glass test device includes three separate microchannels with 0.04 cm diameter and 10 cm length. In this work, the carbonate calcium powder and distilled water were used for the suspension preparation. Based on the carbonate calcium powder catalog, pH is equal to 9.5, mean particle size is equal to 2 microns, CaCO3 content is equal to 98%, MgCO3 content is equal to 1.5%, Fe2O3 content is equal to 0.2%, and insoluble HCl content is equal to 0.15%.

#### Core flooding tests

##### Materials

*Brine*. In this work, both injected water and formation water were prepared as synthetic using analytical grade salts of NaCl, NaHCO_3_, Na_2_SO_4_, CaCl_2_.2H_2_O, MgCl_2_.6H_2_O, SrCl_2_.6H_2_O with high purity from Merck company. This was accomplished by dissolving a precise quantity of each salt in deionized water, stirring the brine solutions for 24 h, and then filtering the brine solutions using paper filters. Therefore, the final brine solutions will be stable and free of suspended scales. Based on the data that are currently available from the examined oil field, Table 3 in this work establishes the chemical composition of seawater from the Persian Gulf as injected water and formation water.

**Table 3:** Chemical composition of Persian Gulf seawater and formation water.

Composition	Na^+^ (mg/L)	Ca^2+^ (mg/L)	Mg^2+^ (mg/L)	Cl^−^ (mg/L)	Sr^2+^ (mg/L)	SO_4_^2−^ (mg/L)	HCO_3_^−^ (mg/L)	pH (mg/L)	TDS (mg/L)	Ionic strength (mol/L)
Seawater	16,814.54	596.5	1945	29,334	0	4360	184.5	8.15	53,235	1.06
Formation water	59,112.8	23,547.4	2744.4	141,151.25	976	396.2	294	6.16	228,222	4.71

**Figure 8:**
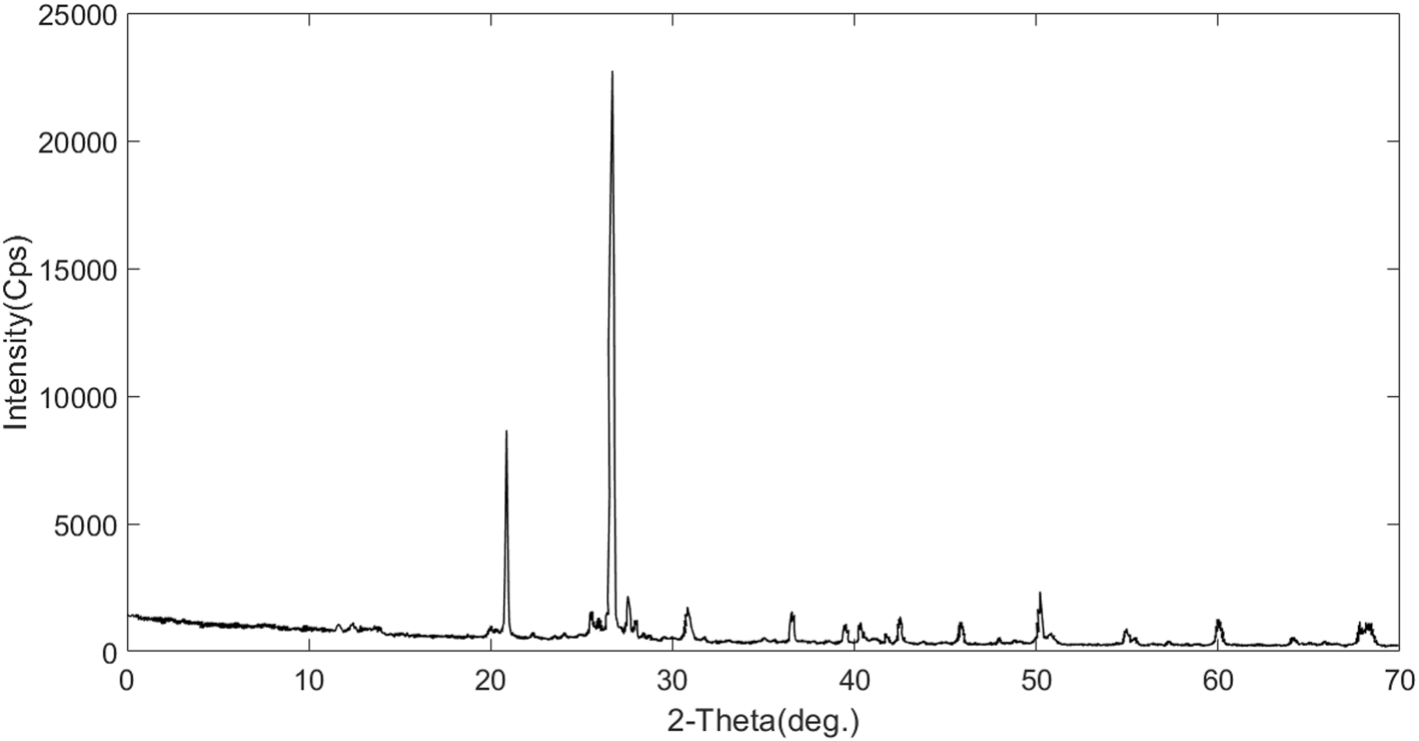
XRD result of core sample.

**Figure 9:**
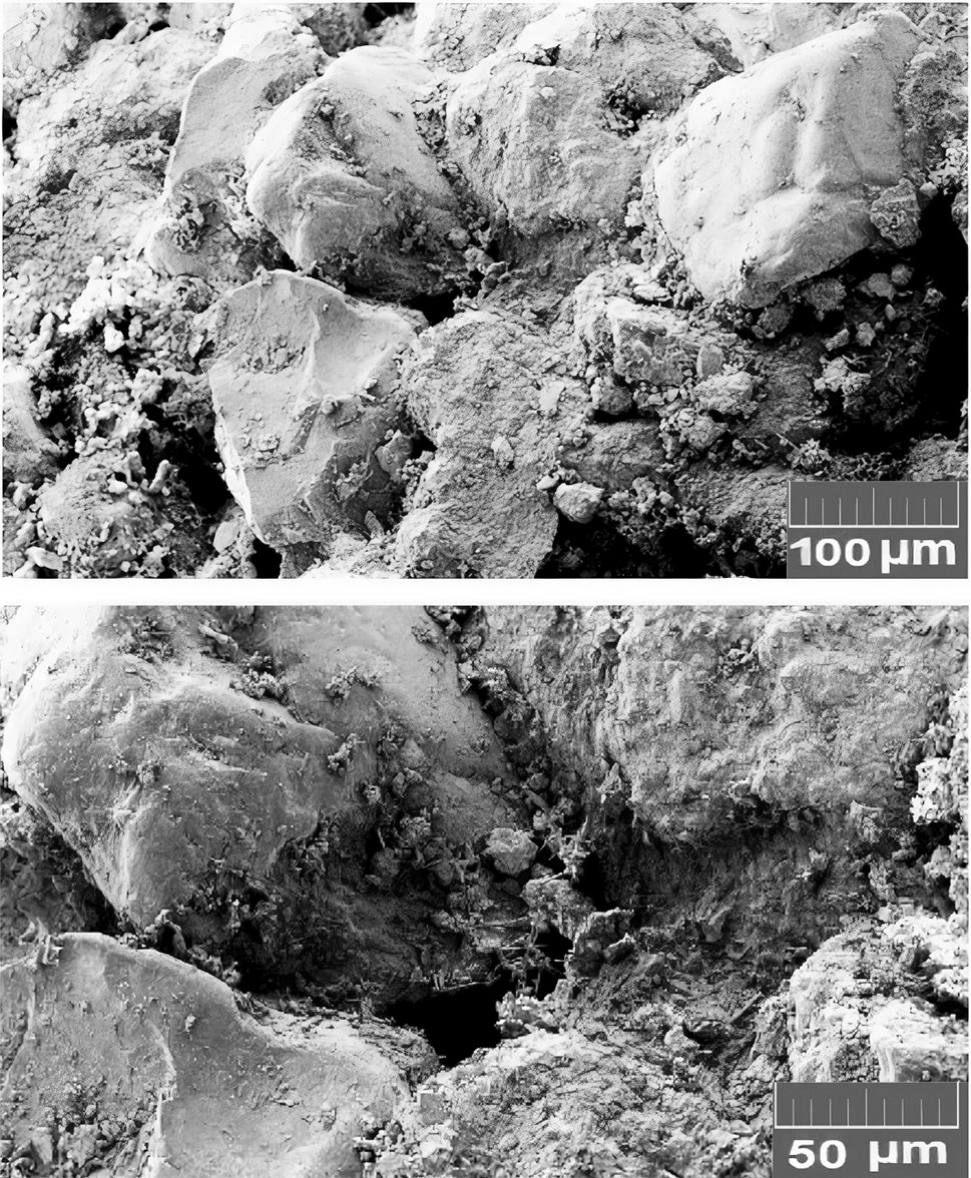
SEM images of core sample.

*Rock sample*. Based on core mineralogy from the XRD test and SEM images analyses, the rock is made of quartz grains with carbonated cements. Figures 8 and 9 show the XRD results and SEM images of the core. Mineralogical data from XRD analyses are given in Table 4.

**Table 4:** Core sample mineralogy based on XRD results.

Phases	Quartz	Dolomite	Microcline	Anorthite	Kaolinite	Gypsum	Total
Percentage %	66.43	19.48	5.42	4.16	2.34	2.17	100

##### Experimental procedure

**Figure 10:**
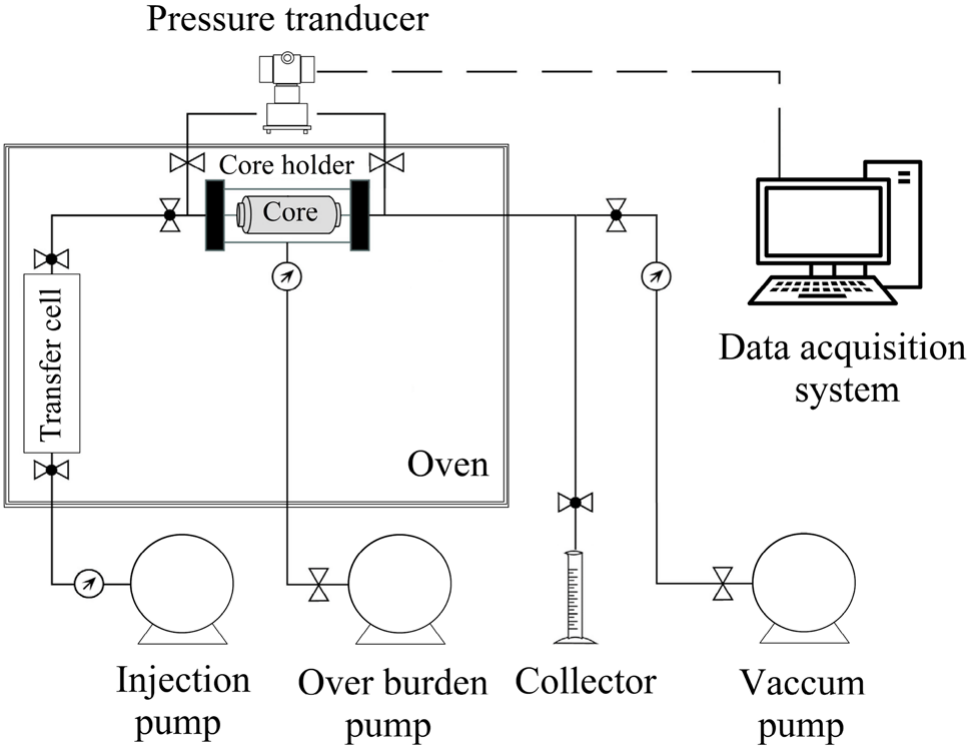
Schematic of core flooding apparatus.

Three core samples were taken from an Iranian sandstone reservoir for core-flooding tests. All the core samples before seawater flooding were first flooded with 1000 ppm NaCl until 4 PV to remove any dissolvable salts. After that, they were cleaned with a Soxhlet extractor device using toluene at 23 °C, dried in an oven at 100 °C, and left alone for 24 h. In Table 5, the core samples' physical characteristics are listed. Figure 10 depicts the core flooding system's schematic.

**Table 5:** Core samples physical properties.

No	Length (cm)	Diameter (cm)	Pore volume (cm^3^)	Porosity (%)	Absolute permeability (md)
1	13.3	3.8	22.85	18.05	49.73
2	13.3	3.8	24.32	17.52	46.84
3	13.3	3.8	23.81	17.13	47.76

##### Results and analysis

**Figure 11:**
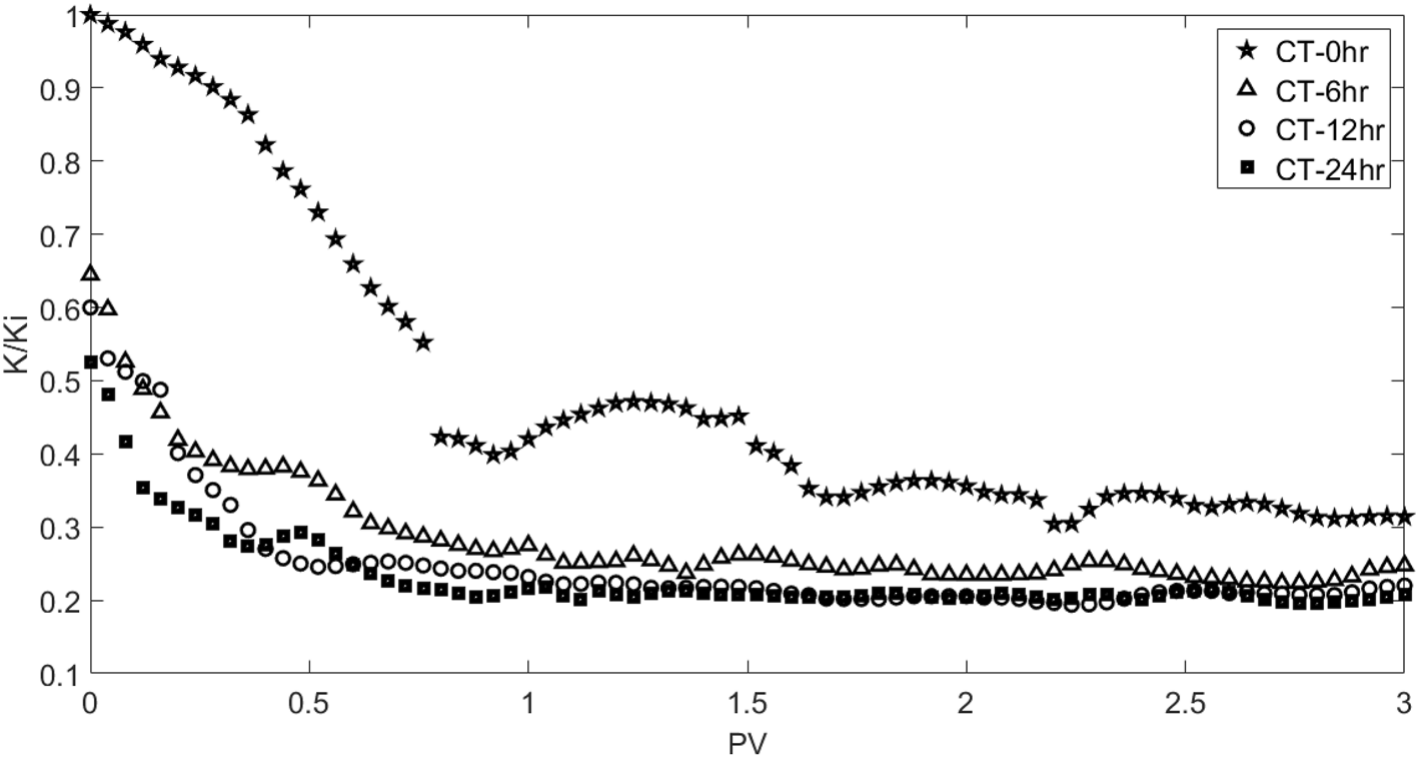
Variation of permeability ratio, showing effect of contact time during seawater flooding.

In this section, synthetic seawater was injected into the core at a continuous flow rate of 0.2 mL/min at reservoir conditions, which are a temperature of 85 °C and pressure of 3222 psi, following core preparation, which comprises cleaning and saturation of the core with synthetic formation water. Seawater flooding was implemented in four scenarios including three steps of seawater injection until 2 pore volumes and then the core was set aside for 0, 6, 12, and 24 h and injection of seawater until 3 pore volumes at the end. The injection of seawater was stopped in this study to investigate the effect of the amount of time formation water had to touch the seawater. According to Fig. 11, the ratio of final permeability to beginning permeability as a function of injected pore volume was shown in order to evaluate the contact time during seawater injection. The permeability of the core after seawater flooding was determined using Darcy's linear flow equation.

**Figure 12:**
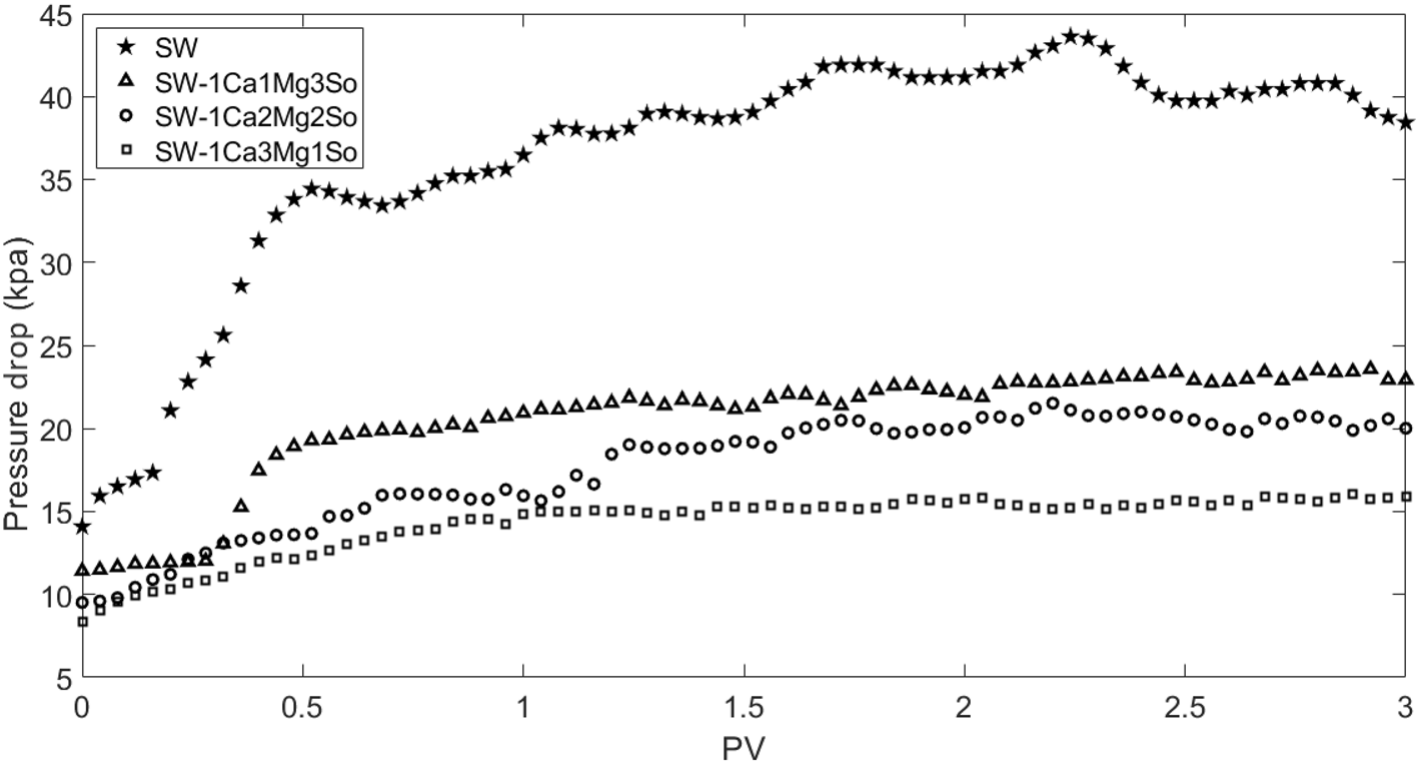
Pressure drop as function of injected pore volume for different modified seawaters.

**Figure 13:**
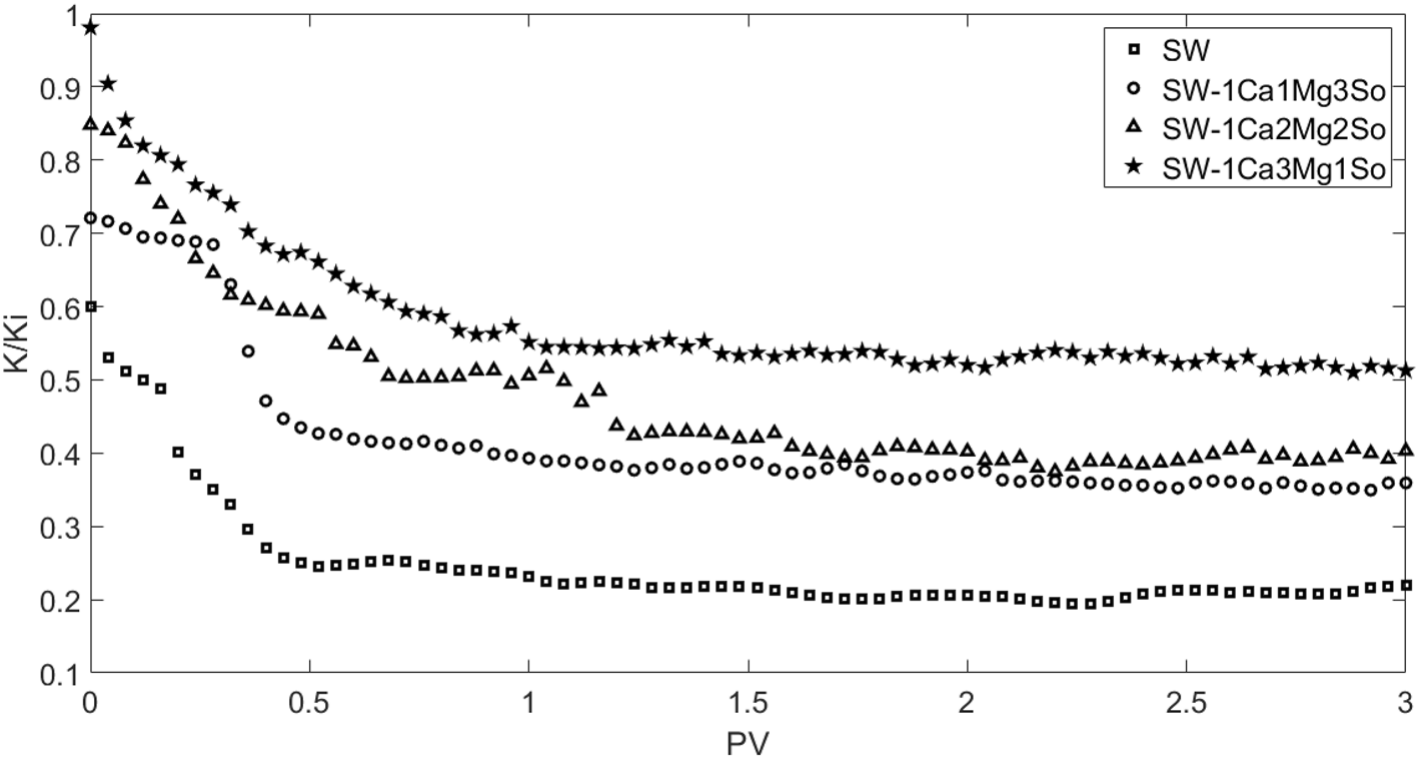
Permeability ratio as function of injected pore volume for different modified seawaters.

Based on Fig. 11, the permeability damage increased with an increase of contact time, but after 12 h, reduction of permeability was not significant. Therefore, in this work, after core preparation, in each scenario of seawater flooding, 2 pore volumes of modified seawater were initially injected, and then injection was stopped for 12 h, and 3 pore volumes of modified seawater were injected at the end. Figures 12 and 13 display the pressure drop and permeability ratio as a function of injected pore volume for flooding the seawater from the Persian Gulf and different modified seawaters.

Generally, the permeability damage mechanism involved in scale nucleation, precipitation, growth, and deposition in rock pores. The scale nucleation and precipitation are faster than scale growth and deposition. According to Figs. 11, 12, and 13, for example in the case of pure seawater injection (SW), the maximum pressure drop and consequent permeability decrease occur until 0.5 PV, then the permeability decrease becomes smoother. Thus, permeability decreases involves two periods. In the initial period, the main reason for permeability decrease is pore clogging due to scale nucleation and precipitation, which leads to a sharp decrease, but in the later period, the main reason for permeability decrease is pore blocking due to scale growth and deposition, which leads to smooth decrease. Therefore, the fluctuation of pressure drop in the initial period is lower than the later period. The reason for pressure drop fluctuation is pore blocking due to bridging (jamming) and bridge breaking due to supplied pressure.

Based on scale precipitation prediction by OLI ScaleChem software and core flooding experiment results, an increase in magnesium ion concentration or decrease in sulfate ion leads to lower scale deposition and finally lower permeability damage. Therefore, the case of SW5D-1C3M1S is the smart water in our case study.

### Digital core analysis and pore network generation

In this study, the watershed segmentation algorithm (WA) was used to create 2-D binary images of the core thin section, and ImageJ software was used to quantify parameters of the porous media structure, such as porosity, pore size distribution, and throat size distribution. Utilizing noise filtering and majority transform techniques, the watershed segmentation algorithm (WA) helps to prevent small, unconnected pores^19–23^.

**Figure 14:**
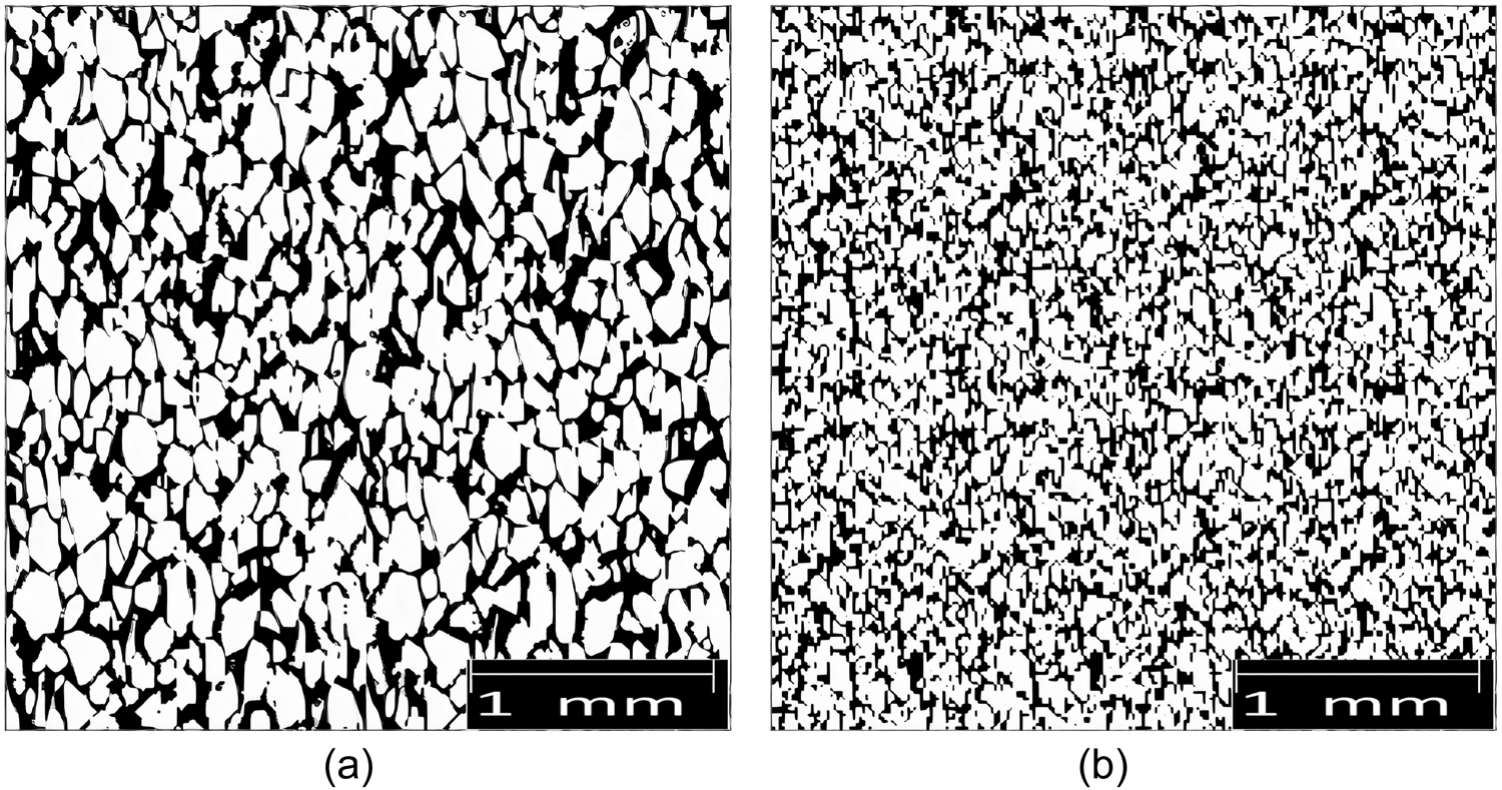
2-D binary images of core thin sections: (**a**) before seawater flooding and (**b**) after seawater flooding.

In this study, the watershed segmentation algorithm is enhanced with a city-block distance transform and median filtering to more effectively identify and distinguish overlapping porous geometries. Pore size distribution was calculated by measuring the area of detected pores and computing the radius of corresponding circles with the same area. Porosity was calculated by dividing the area summation of detected pores across the entire 2-D picture area. Additionally, the area of detected throat bodies was measured, and the radius of equivalent cylinders with the same area was calculated in order to determine the throat size distribution based on the watershed ridge line, which is the contact line between two neighboring pores, and the coordination number, which is the average number of independent throats connected to a particular pore. Figure 14 displays the 2-D binary images generated from SEM image binarization that were taken before and after seawater flooding.

Based on Fig. 14, the total porosity of 2-D binary images was calculated by dividing the area summation of pore space over the total 2-D image area using ImageJ software. For this purpose, the first images of rock were converted into an 8-bit image to enhance the image contrast. Then, analysis was done after elimination of random noises and segmentation of the image. Eventually, the value of total porosity was 31.12% and 22.76% before and after seawater flooding which was related to both interconnected and isolated pore space. Therefore, the amount of deposited scales in the core by subtraction of total porosity after seawater flooding from total porosity before seawater flooding can be calculated equal to 8.36% and this amount can be considered an approximate of mobile particle concentration in pore space.

**Figure 15:**
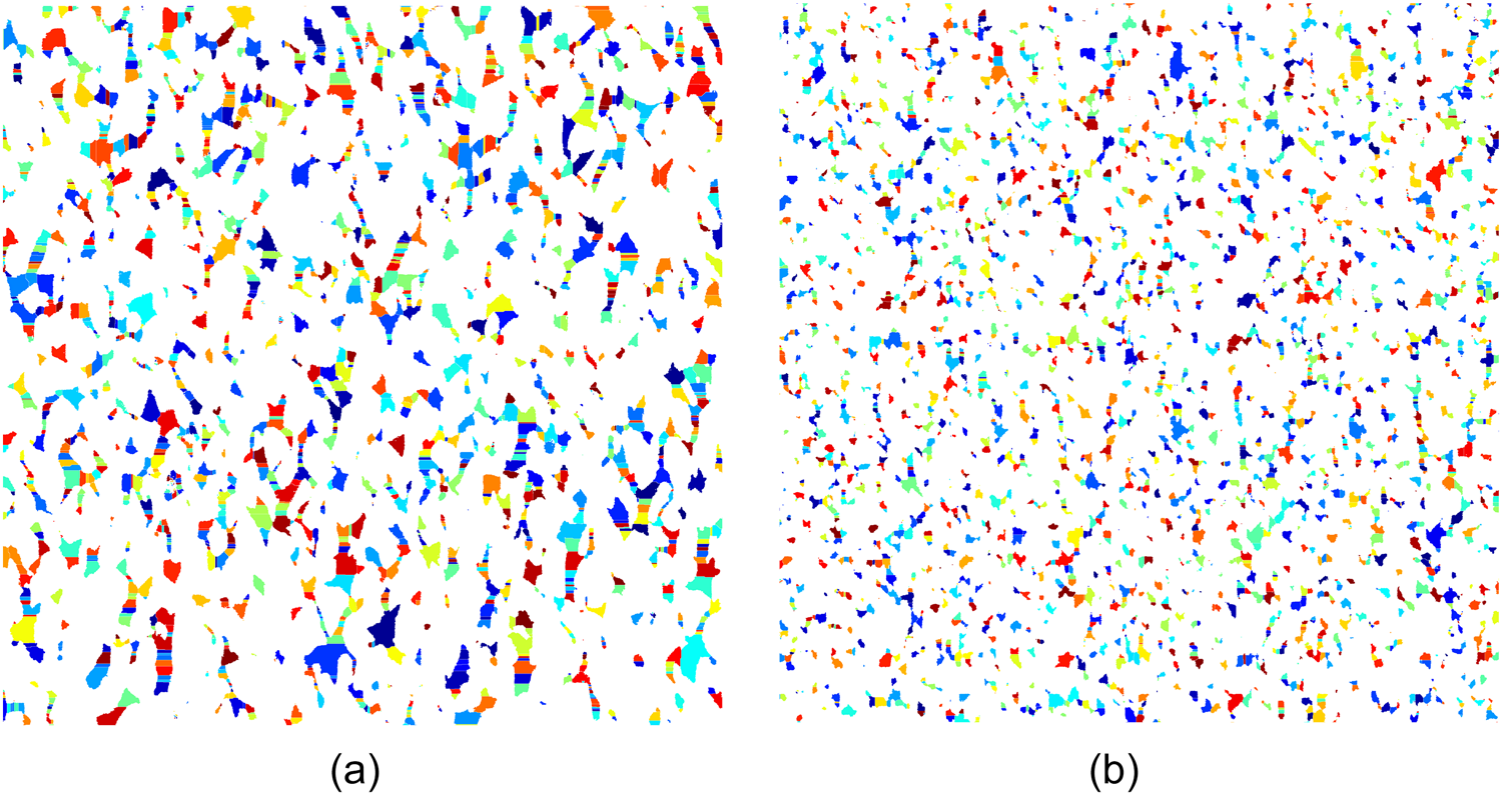
2-D pore space segmentations: (**a**) before seawater flooding and (**b**) after seawater flooding.

Figure 15 shows the pore space segmentations before and after seawater flooding which are obtained after applying the watershed segmentation algorithm on 2-D binary images. The calculated effective porosity value of 2-D images by the watershed segmentation algorithm was 18.34% and 10.93% before and after seawater flooding which show about 5% error related to the experimental measurement.

**Figure 16:**
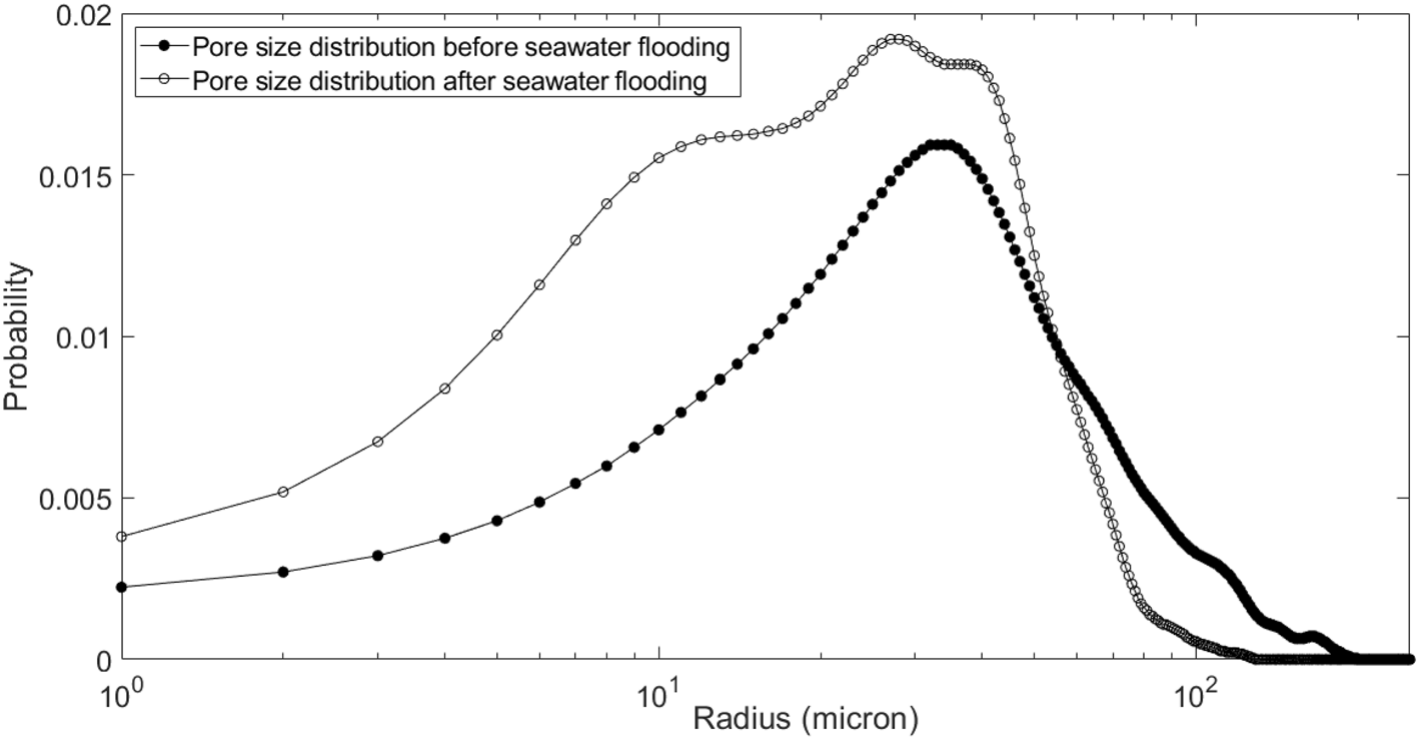
Pore size distribution before and after seawater flooding.

**Figure 17:**
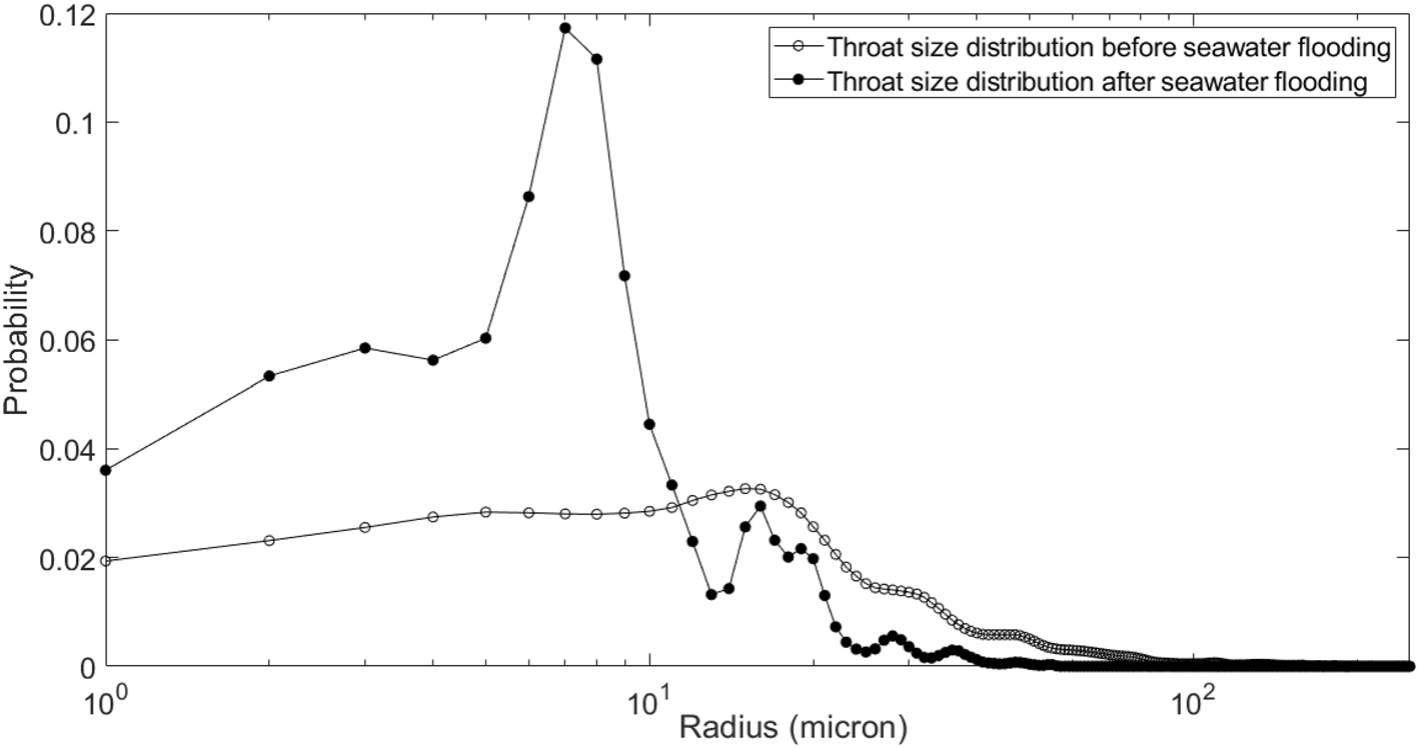
Throat size distribution before and after seawater flooding.

Figures 16 and 17 show the pore size distribution and throat size distribution before and after seawater flooding, which are validated by the porosity match.

**Figure 18:**
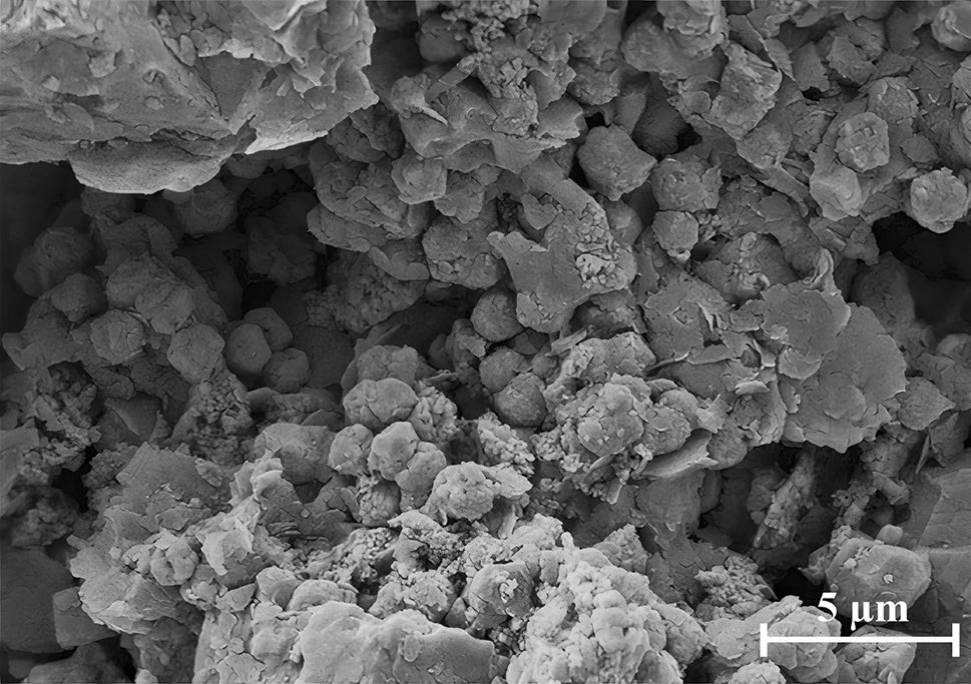
SEM image of rock pore space after seawater flooding.

**Figure 19:**
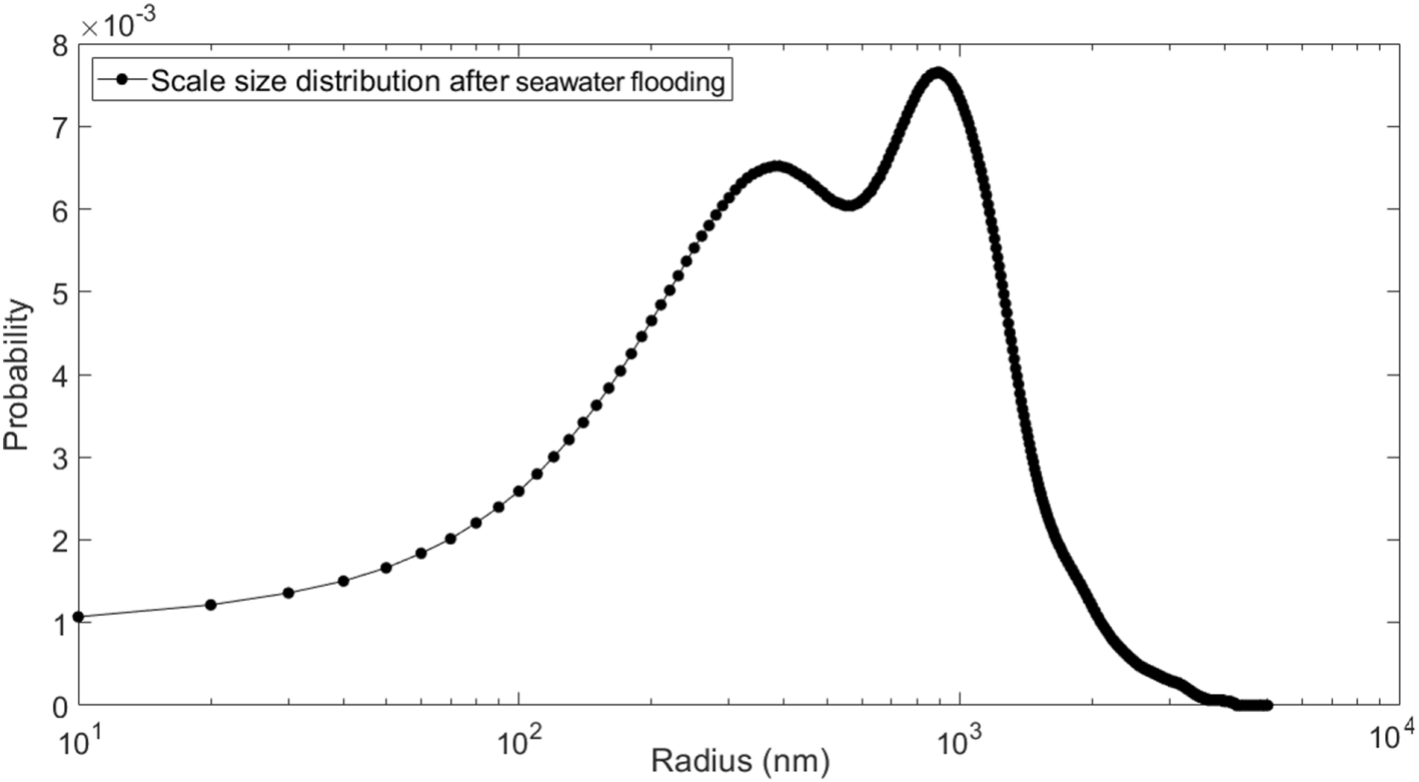
Scale size distribution after seawater flooding.

In this work, size distribution of formed scales in the rock pore space was determined after seawater flooding using ImageJ software. Figures 18 and 19 show the SEM image of rock pore space and scale size distribution after seawater flooding, respectively.

**Figure 20:**
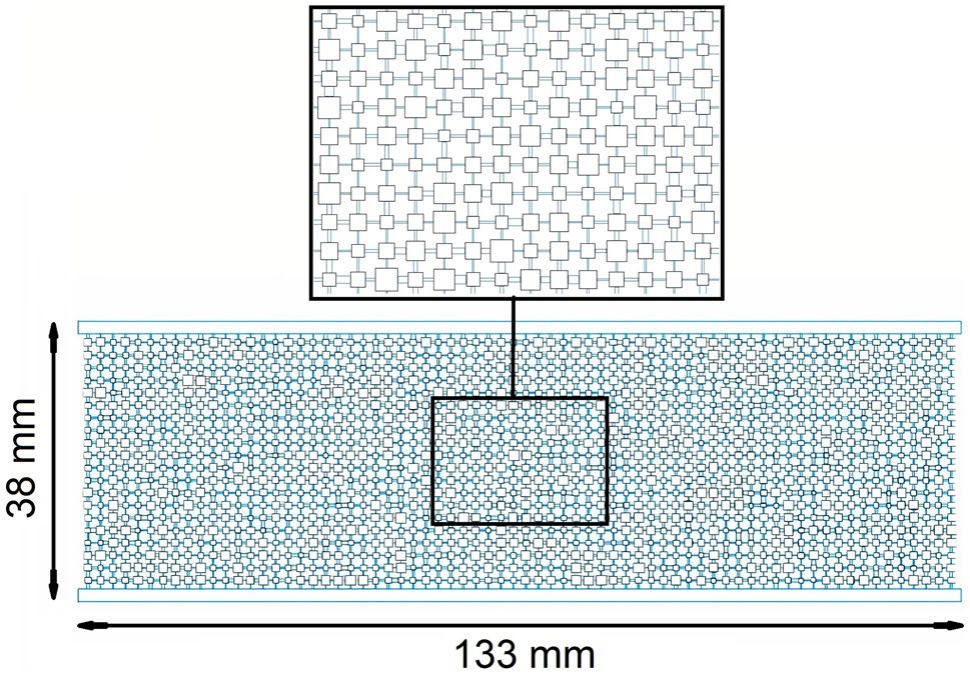
2-D generated pore network according to core dimension.

In this work, the 2-D pore network was generated based on porosity, pore size distribution, and throat size distribution as random distribution. Table 6 shows the values of different parameters used to generate the pore network. According to core dimension, the 2-D generated pore network has a total pore number of 1400 and total throat number of 1380 and in the pore network, pores were considered as squares instead of circles as shown in Fig. 20.

**Table 6:** Parameters used to generate pore network as random distribution.

Parameter	Minimum pore size	Maximum pore size	Mean pore size	Minimum throat size	Maximum throat size	Mean throat size	Porosity
Value	2 µm	366 µm	67 µm	2 µm	144 µm	31 µm	18.34%

### Numerical modeling

The method created by Bagrezaie et al. was applied in this study to simulate the injection of seawater into core samples, which involves the flow of seawater and the formed solid scales^24^. This approach is summarized as follows:

The DPM model's simulation of solid particles and fluid flow in a single microchannel (pore/throat).

Development of a proxy model based on the results of simulation of the flow in a single microchannel.

Dual pore scale model simulation of particle-fluid flow in the pore network (core).

#### DPM model development

The DPM model created by Bagrezaie et al. was utilized to simulate particle-fluid flow in each pore and throat. They modified the default DPM model of Ansys Fluent software for laminar and single-phase flow of distillated water and particles in the microchannel. As a result, the modeling did not take into account the impacts of particle breakup, capillary forces, van der Waals forces, and electrostatic forces. The modified DPM model created by Bagrezaie et al. was added to in this work, along with the impacts of van der Waals forces and electrostatic forces, although other presumptions remained^24^.

The general DPM model mathematical formulation is available in the literature^24^. The main mechanisms of permeability damage in this work are particle detachment from the rock surface, particle attachment to the rock surface, particle agglomeration, and clogging and/or blocking of pore throats. These mechanisms are based on the physics of scale movement by seawater in rock pore space. As a result, UDF code should be used to add the impacts of these mechanisms to the Ansys Fluent software's generic DPM model. We adopt the Bagrezaie et al. approach, which proposed a critical velocity concept based on semi-empirical correlation for particle-fluid flow in the microchannel as follows, to apply the effects of particle detachment and attachment in pore and throat media^24^:

1 $${V}_{c}=\frac{D}{8}\frac{{\tau }_{w} }{\mu }$$

2 $${\tau }_{w}=163\left(\left(\frac{{\rho }_{p}}{\rho }\right)-1\right)\rho {d}_{p}$$

In Eq. (1), ${V}_{c}$ is the critical velocity and defined as the velocity above which particles are detached from the wall and is applicable for stable laminar flow, ${\uptau }_{w}$ is the wall shear stress, and D is the microchannel diameter. Therefore, the particles become detached from the wall surface if the particle adhesion forces cannot overcome the fluid forces. According to research on various particle detachment mechanisms, the rolling and sliding mechanisms particularly the rolling mechanism, which predominates for spherical particles are the principal causes of particle detachment^25^. The rolling mechanism states that the particle begins to roll if the moment owing to fluid forces at the interface of the particle–wall contact exceeds the moment due to particle adhesion forces. As a result, rolling will cause the particle detachment condition to be as follows:

3 $${F}_{D}\left(\frac{{d}_{p}}{2}-b\right)+{F}_{L}\left(a\right)\ge {F}_{ad}\left(a\right)$$

**Figure 21:**
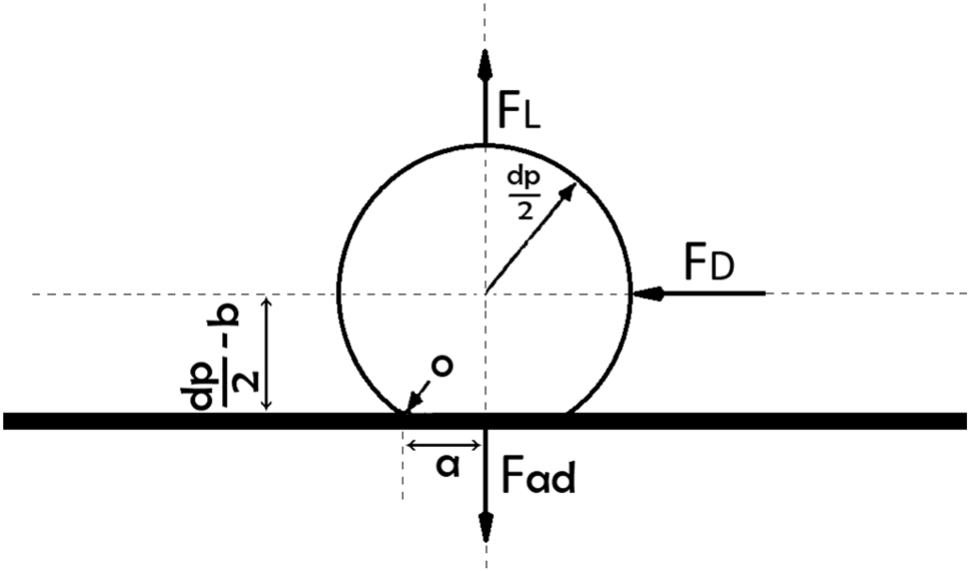
Geometric attributes of a spherical particle in contact with a surface (Bagrezaie et al.^24^).

In Eq. (3), ${F}_{D}$ is the drag force, ${F}_{L}$ is the lift force, ${F}_{ad}$ is the particle adhesion force, $b$ is the particle deformation normal to surface, and ${\varvec{a}}$ is the particle deformation along the surface as shown in Fig. 21.

The impact of lift force on particle separation is typically far less than that of drag force in actual situations. Also, in the elastic particle adhesion, b is very small in comparison with particle radius. Therefore, Equation (3) is simplified to:

4 $${F}_{D}\left(\frac{{d}_{p}}{2}\right)\ge {F}_{ad}\left(a\right)$$

However, due to the salinity of the water, the effects of van der Waals and electrostatic forces during seawater injection are considerable. The London-van der Waals force, which tends to be stronger than other van der Waals forces, and the electrical double layer force, which only manifests itself for small particles with a diameter of less than 5 microns, were taken into account in this study^26–28^. The London-van der Waals force and the electrical double layer force both have an effect in the direction normal to the surface, but the London-van der Waals force is invariably attracting while the electrical double layer force is often repulsive in the presence of water^29^. Equation (4) can therefore be represented as:

5 $${F}_{D}\left(\frac{{d}_{p}}{2}\right)+{F}_{EL}\ge {F}_{ad}\left(a\right)+{F}_{VDW}$$

In equation (5), drag force on a spherical particle can be calculated as follows:

6 $${F}_{D}=\frac{3\pi }{2}{{d}_{p}}^{2}\rho {{u}^{*}}^{2}$$

where ${{\varvec{u}}}^{\boldsymbol{*}}$ is the wall shear velocity and calculated as follows:

7 $${u}^{*}=\sqrt{\frac{2\mu V}{\rho {d}_{p}}}$$

In equation (5), electrical double layer force is calculated as follows^30^:

8 $${F}_{EL}= \frac{2\pi \epsilon {\epsilon }_{0} ({{\zeta }_{p}}^{2}+{{\zeta }_{\mathsf{g}}}^{2})\kappa {e}^{-\kappa \delta }}{1-{e}^{-2\kappa \delta }}\left(\frac{2{\zeta }_{p}{\zeta }_{\mathsf{g}}}{{{\zeta }_{p}}^{2}+{{\zeta }_{\mathsf{g}}}^{2}}-{e}^{-\kappa \delta }\right)$$

where є is the relative dielectric constant of water, ${\upepsilon }_{0}$ is the dielectric constant in vacuum, δ is the separation distance between the particle and surface, ${\zeta }_{p}$ and ${\zeta }_{\mathsf{g}}$ are the zeta potentials of the particle and surface, respectively. $\kappa$ is the Debye reciprocal double layer thickness which is calculated as below:

9 $$\kappa = \sqrt{\frac{2 I{{N}_{A}e}^{2}}{\epsilon {\epsilon }_{0}T\mathtt{k}}}$$

where $I$ is the ionic concentration in the water, ${N}_{A}$ is Avogadro’s number, $e$ is the elementary charge, $T$ is absolute temperature, and $\mathtt{k}$ is Boltzmann’s constant.

In equation (5), particle adhesion force based on Hertz contact theory can be calculated as follows:

10 $${F}_{ad}=\frac{3}{4}\pi {W}_{A}{d}_{p}$$

In Eq. (10), ${W}_{A}$ is the thermodynamic work of adhesion. The deformation of particle along the surface is calculated as:

11 $$a=\frac{3}{2}\pi \frac{{W}_{A}{{d}_{p}}^{2}}{{K}_{c}}$$

12 $${K}_{c}=\frac{4}{3}{\left[\frac{\left(1-{{\vartheta }_{s}}^{2}\right)}{{E}_{s}}+\frac{\left(1-{{\vartheta }_{p}}^{2}\right)}{{E}_{p}}\right]}^{-1}$$

where ${K}_{c}$ is composite Young’s modulus, ${\vartheta }_{s}$ and ${E}_{s}$ are Poisson’s ratio and Young’s modulus value of the wall surface, respectively, and ${\vartheta }_{p}$ and ${E}_{p}$ are Poisson’s ratio and Young’s modulus value of the particle, respectively.

In equation (5), London-van der Waals force is calculated as below^30^:

13 $${F}_{VDW}=\frac{1}{12} \frac{H{{d}_{p}}^{3}}{{{\delta }^{2}\left(\delta +{d}_{p}\right)}^{2}}$$

where $H$ is the Hamaker constant which is expressed for particle-water–sand as follow^31^:

14 $$\mathrm{H}=\left(\sqrt{{\mathrm{H}}_{\mathrm{p}}}-\sqrt{{\mathrm{H}}_{\mathrm{w}}}\right)\left(\sqrt{{\mathrm{H}}_{\mathrm{s}}}-\sqrt{{\mathrm{H}}_{\mathrm{w}}}\right)$$

In addition to the rolling mechanism, a particle can detach from the wall surface under the sliding mechanism. The particle detachment condition due to sliding will be as follows:

15 $${F}_{D}+{F}_{EL}\ge {K}_{s}{F}_{ad}+{F}_{VDW}$$

where ${K}_{s}$ is the static friction coefficient between the particle and the wall surface. Finally, the particle will be detached due to the rolling or sliding mechanism if the summation of the drag and electrical double layer forces exceeds the summation of the adhesion and London-van der Waals forces.

When particle–particle interactions outweigh particle-fluid interactions, particle agglomeration is another important mechanism in the transport of scales by saltwater in rock pore space^32^. Applying particle collision and subsequent particle agglomeration using O'Rourke's algorithm was done in this work. This algorithm considers the particle collision as stochastic and assumes the collision probability as follows:

16 $${P}_{r}=\frac{\pi {\left({r}_{1}+{r}_{2}\right)}^{2}{v}_{rel}{\Delta }_{t}}{{V}_{cp}}$$

In Eq. (16), ${r}_{1}$ and ${r}_{2}$ are the radius of the two particles that participate in the collision, ${v}_{rel}$ is the relative velocity of the two particles, ${\Delta }_{t}$ is the time step used to integrate the trajectories of the particles, and ${V}_{cp}$ is the volume of the continuous-phase cell.

When two particles collide, O'Rourke's algorithm determines if coalescence will occur as a result. If not, the two particles preserve their original physical characteristics, with the exception of their velocities. If the critical offset value is bigger than the actual collision parameter, then the coalescence is the result of collision^33^. Following is a calculation of the actual collision parameter:

17 $${b}_{act}=\left({r}_{1}+{r}_{2}\right)\sqrt{Y}$$

where $Y$ is a random number between 0 and 1 and the critical offset is calculated as follows:

18 $${b}_{crit}=\left({r}_{1}+{r}_{2}\right)\sqrt{\mathit{min}\left(1.0,\frac{2.4 f}{{W}_{e}}\right)}$$

In Eq. (18), ${W}_{e}$ is the collisional Weber number and ${\varvec{f}}$ is a function of the relative radius and is calculated as follows:

19 $$f\left(\frac{{r}_{1}}{{r}_{2}}\right)={\left(\frac{{r}_{1}}{{r}_{2}}\right)}^{3}-2.4{\left(\frac{{r}_{1}}{{r}_{2}}\right)}^{2}+2.7\left(\frac{{r}_{1}}{{r}_{2}}\right)$$

**Figure 22:**
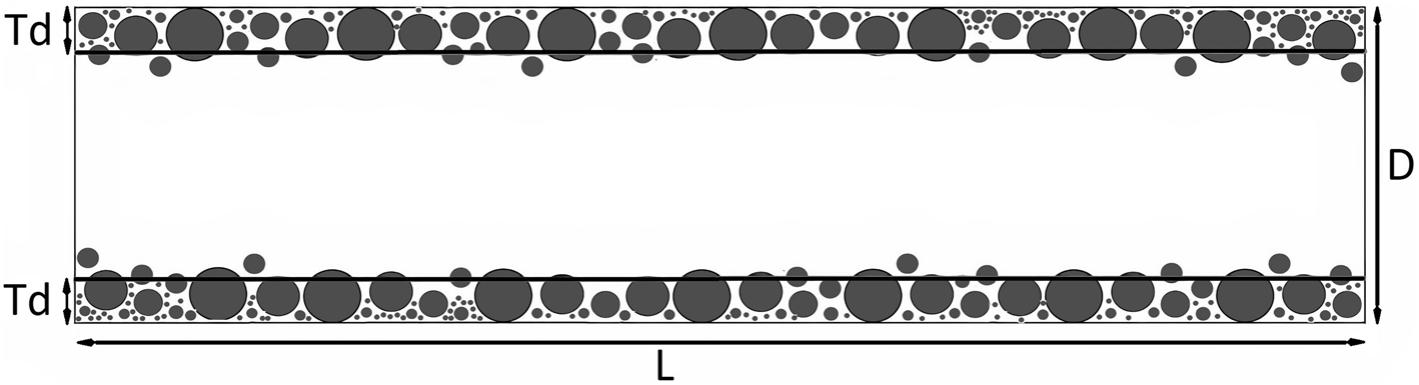
Average thickness of deposited particle layers on microchannel walls (Bagrezaie et al.^24^).

In this study, the dynamic mesh option of Fluent software was used to apply the effects of the pore throat clogging mechanism by microchannel wall motion to the center of the microchannel in the model. By adjusting the wall's velocity, the decrease in microchannel cross-sectional area was taken into account based on the average thickness of the deposited particle layer in the preceding time steps. The average thickness of the deposited particle layer on each wall of the microchannel in the 2-D model can be determined using the formula below^24^:

20 $$Td=\frac{\sum_{i}^{n}{A}_{pi}}{L}$$

21 $$Ap=\pi {r}^{2}$$

where ${\varvec{n}}$ is the total number of deposited particles on the wall in each time step, ${\varvec{A}}{\varvec{p}}$ is the area of deposited particle and ${\varvec{L}}$ is the length of microchannel as shown in Fig. 22.

By setting the outlet boundary condition to no flow boundary, the impacts of the pore throat blocking mechanism are also implemented in the mentioned model. In this work, the pore throat blocking mechanism will be initiated when the free cross-section available for fluid flow is lowered to 40% of the initial value, based on the range of Reynolds numbers and particle concentration^34,35^.

#### Simulation of scales motion in pore space by modified DPM model

**Figure 23:**
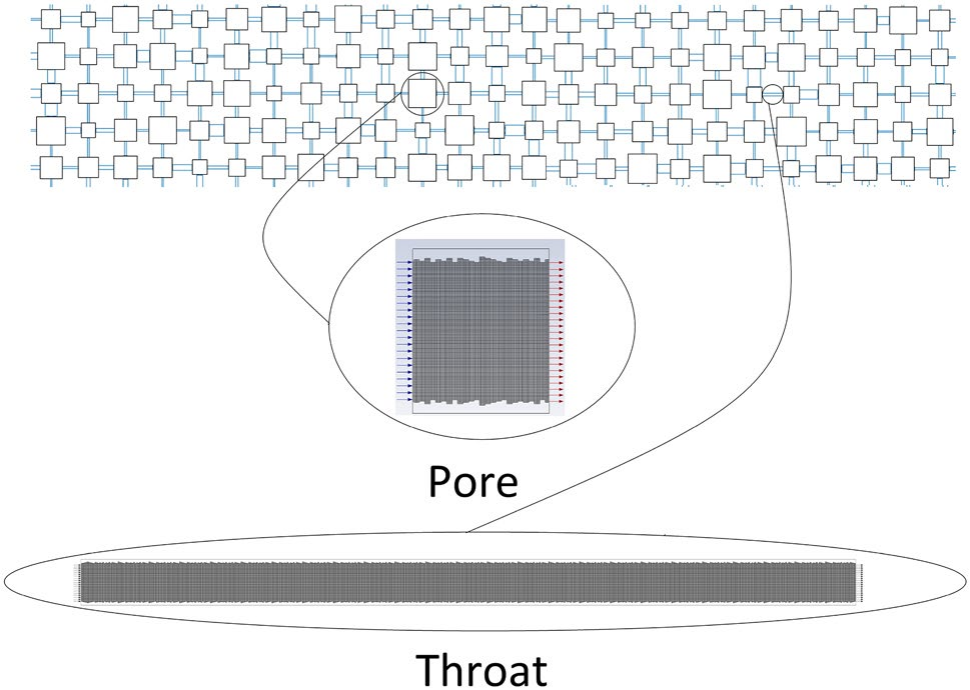
Schematic of 2D geometric models of microchannel.

The software SolidWorks 2018 was used to create several 2-D geometric models with two-dimensional surface roughness profiles. In this work, pores were assumed to be square microchannels and throats to be rectangle microchannels. Due to the heterogeneity impact, the random rectangular surface roughness model was chosen among the other surface roughness models. Figure 23 depicts a schematic of the pore network and 2-D geometric models, which also incorporate the random rectangular surface roughness model. The grid independence test based on the Poiseuille number and Ansys meshing software were used to mesh geometric models with a uniform quadratic mesh^36^.

The prenominated DPM model is used in this study to solve the governing equations for simulating the movement of scales and seawater in a single microchannel using Fluent 19 CFD software. For precise calculation of objective functions in the simulations, the pressure-based solver and the gravity effect are taken into account. Water was characterized in the material option as having characteristics resembling those of seawater and calcium carbonate was selected in the material option. Additionally, a logarithmic Rosin-Rammler function was used to generate a scale size distribution with a size range of 5 nm to 5000 nm. The diffusion-based smoothing method was chosen in the dynamic mesh option. The QUIK scheme was used to implement the suspension volume fraction, the SIMPLE method was used to relate velocity and pressure, and the Second Order Upwind approach was used to interpolate additional parameters in the equations. Upper and lower edges of the microchannel were taken into account as walls in the boundary condition section, the inlet boundary as velocity inlet and the outlet boundary as outflow^24,37^.

Finally, several scales-seawater flooding scenarios were simulated up to three pore volumes in a single microchannel, and in each scenario, pressure drop was calculated as a function of injected pore volume. The range of various parameters in several simulated scenarios is displayed in Table 7.

**Table 7:** Range of various parameters in the different simulation scenarios.

Parameter	Value
particle concentration (PC)	6 to 10%
Ratio of particles mean diameter to microchannel diameter (P/D)	0.001 to 0.2
Ratio of microchannel length to microchannel diameter (L/D)	1 to 250
Reynolds number (Re)	0.3

#### Proxy model development

In engineering problems with complex physics, numerical simulation is computationally expensive due to solving the governing equations by performing a large number of simulation runs. Therefore, development of a proxy model is at the forefront of engineering problem solving and optimization. The application of the proxy model in petroleum engineering has been made in the areas of risk analysis, reservoir characterization, upscaling geologic models, production optimization, field development planning, history matching, and flow simulation in porous media^38–41^. Machine learning and pattern recognition are used to operate the proxy model based on system behavior. Artificial neural networks (ANN) are applicable as a virtual intelligence technique for identifying and approximating the relationship between inputs and outputs of a system that is highly non-linear. Proxy model development by various techniques, such as polynomial, radial basis function, Gaussian process, ANN, and genetic algorithms, is common^42^. Three processes are involved in creating a proxy model using artificial neural networks: data collection based on experimental findings or simulation outcomes; network training based on error feedback to the network; and network validation^24^.

Based on simulation results of scale-seawater injection into a microchannel using the modified DPM model, a proxy model was created in this work utilizing the artificial neural network (ANN) method. Additionally, for data collection, injected pore volume as an input parameter, particle concentration, microchannel length to microchannel diameter ratio, particle mean diameter to microchannel diameter ratio, and pressure drop as an output parameter were all used.

**Figure 24:**
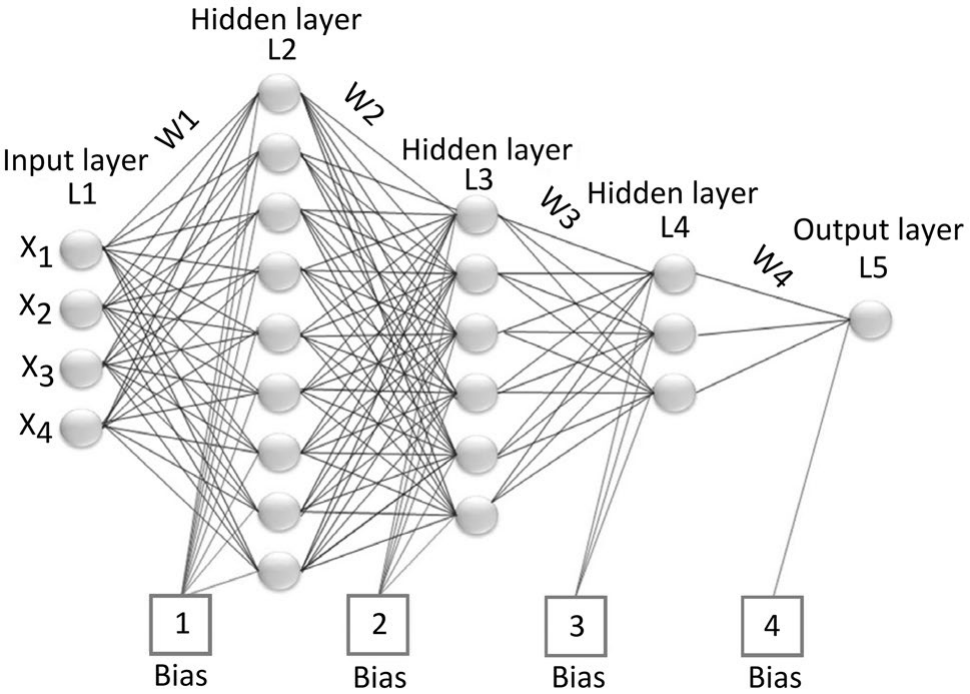
Optimum network architecture.

The utilized artificial neural network includes three hidden layers with, respectively, 9, 6, and 3 neurons (Fig. 24). Through the MATLAB Neural Fitting Toolbox, the ANN is trained using the Levenberg–Marquardt backpropagation technique. In order to achieve the lowest mean squared error, we trained the network 653 times. Seventy percent of the 14400 dataset samples were used for network training, fifteen percent for network verification, and fifteen percent for network testing.

#### Dual pore scale model development

The first introduction of pore network modeling was made by Fatt in 1956, and it comprises pores that are connected to one another by interconnected throats^43^. This method involves solving a set of basic equations that represent the conservation of mass at each pore in order to derive the pressure field of the network model. Following is a derivation of the fundamental equation for each pore in a single-phase system:

22 $$\sum_{j}{q}_{ij}=0$$

In this equation, ${{\varvec{q}}}_{{\varvec{i}}{\varvec{j}}}$ is the volumetric flow rate of element ${\varvec{i}}$ to a neighbor element ${\varvec{j}}$ and is a function of pressure drop across an element ${({\varvec{P}}}_{{\varvec{j}}}{-{\varvec{P}}}_{{\varvec{i}}})$, the conductivity in element ${{\varvec{g}}}_{{\varvec{i}}{\varvec{j}}}$, and fluid viscosity ${\varvec{\mu}}$, respectively. Therefore, Eq. (22) can be written as follows:

23 $$\sum_{j}[{(P}_{j}-{P}_{j})\frac{{ g}_{ij}}{\mu }]=0$$

By using equation (23) for each element (pore or throat), a system of linear equations is created, and by simultaneously solving this system of equations, the pressure field of the network model is determined^44,45^. Although other approaches, such as Gauss-Seidel, can be used to solve the matrices of a system of linear equations, the developed proxy model was used in this study. Our proxy model can determine the pressure drop across each element (pore or throat) based on the element's characteristics, such as the ratio of the particles' mean diameter to the element's diameter, the ratio of the particles' length to the element's diameter, the particles' concentration, and the injected pore volume.

**Figure 25:**
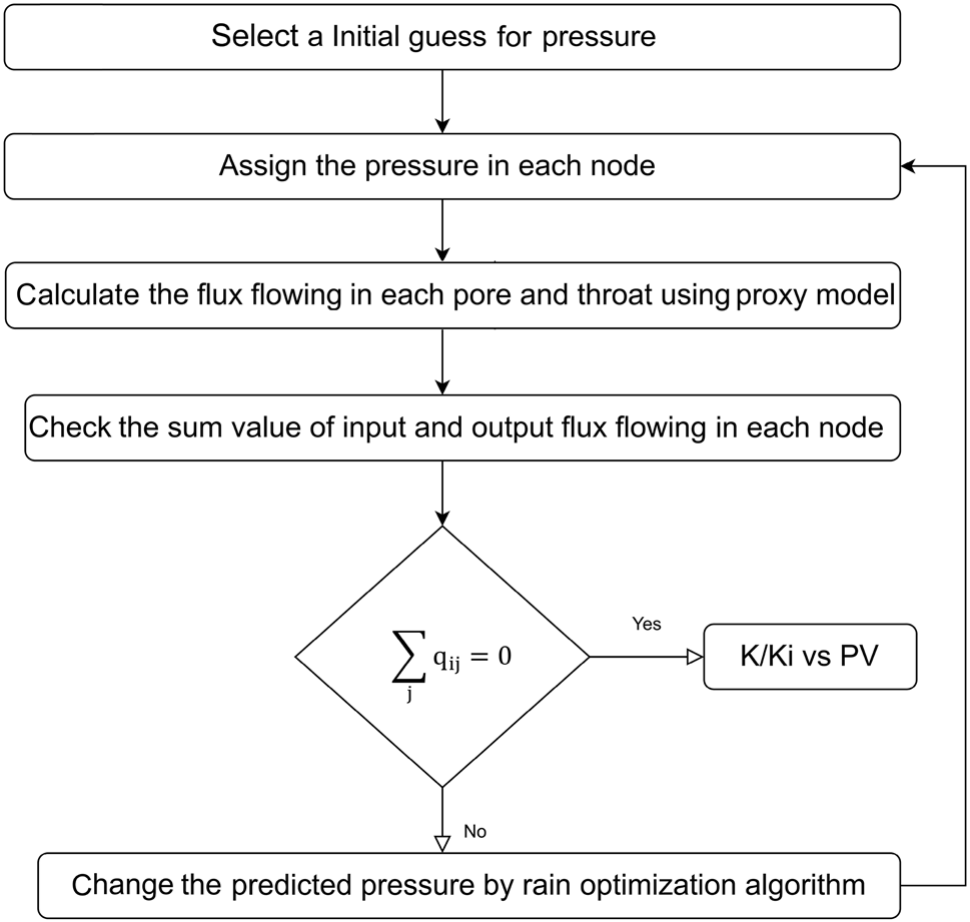
Flowchart for calculating the permeability damage by dual pore scale model.

In this work, a numerical modeling approach to simulation of the scales-seawater movement in a core sample was developed based on the computational fluid dynamic (CFD) and pore network modeling approach using MATLAB software. In this approach, details of flow in the microscopic media (pores and throats) by CFD approach and details of flow in the macroscopic media (core) by pore network modeling approach were analyzed. The pressure decrease brought on by the movement of the scales-seawater in the pore network is first calculated by our dual pore scale model as a function of the injected pore volume. After then, the pressure drop will be converted into permeability damage using Darcy's Law. The flowchart for the dual pore scale model's calculation of permeability damage is shown in Fig. 25.

According to Fig. 25, for calculating the permeability damage by dual pore scale model, first the pressure will be assigned in all nodes of the network model based on initial guess. Then, using the proxy model, the flux going through each pore and throat of the network model will be determined. Following that, the total value of the input and output flux flows at each node will be determined. The problem is solved if the flux sum value for each node is equal to zero; if not, the rain optimization algorithm (ROA), a meta-heuristic approach to problem optimization, would adjust the anticipated pressures^46^. Finally, until the flux sum value for each node equals zero, the aforementioned operations will be repeated repeatedly.

## Validation of modified DPM model

**Figure 26:**
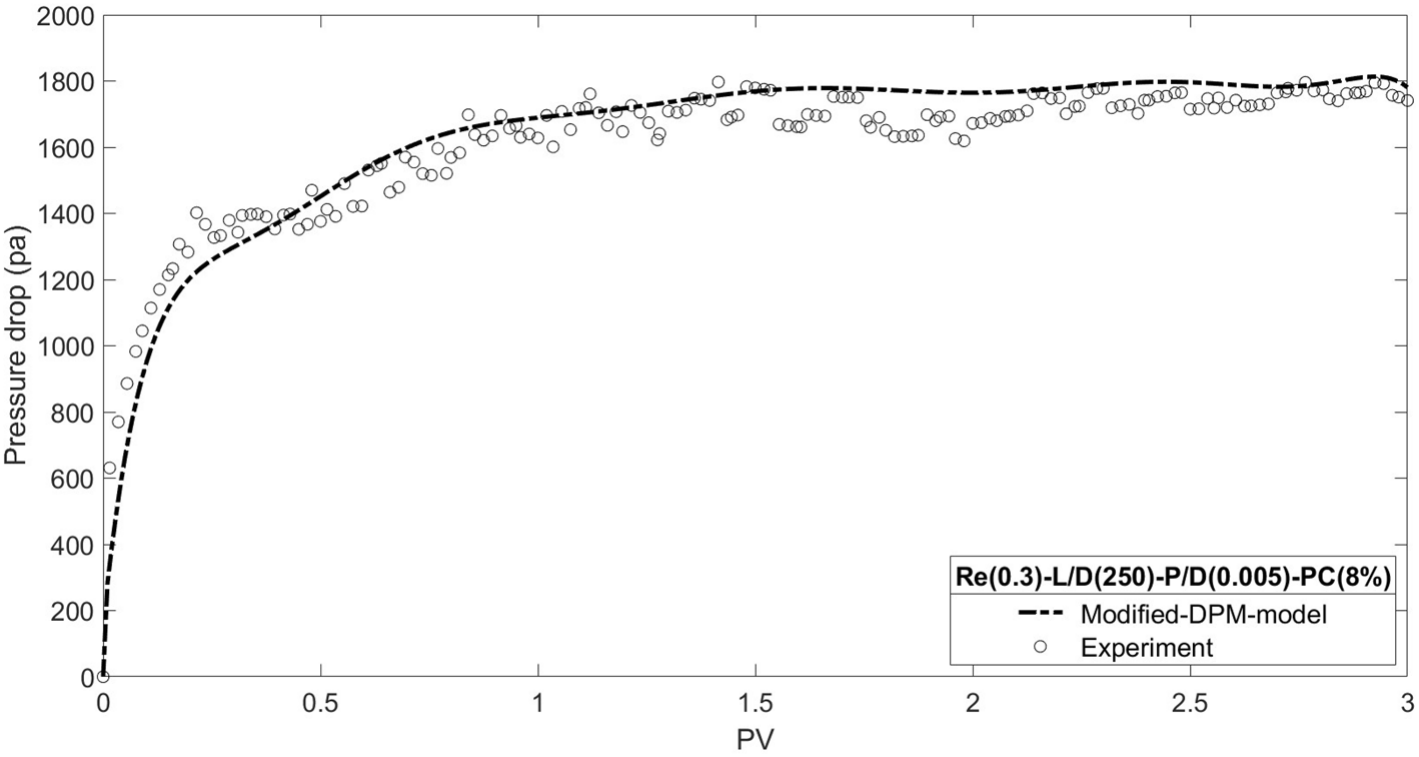
Matching modified DPM model results and experimental data.

Figure 26 shows a good agreement between simulation results using the modified DPM model with experimental data for the base case. In the base case, particles concentration is equal to 8 percent, the ratio of particles mean diameter to microchannel diameter is equal to 0.005, the ratio of microchannel length to microchannel diameter is equal to 250, and the Reynolds number is equal to 0.3.

## Validation of dual pore scale model

For validation of the dual pore scale model, a comparison was made between the simulation results of scales-seawater flow in the network model with experimental data. For this purpose, suspension injection in three particle concentrations including 6, 8, and 10 percent, particles mean diameter equal to 1.8 micron, and Reynolds number equal to 0.3 were simulated according to core flooding experiments. On the basis of Table 8 and the cost function depicted in Equation (23), the initial tuning parameters of the rain optimization algorithm (ROA) are also taken into consideration.

**Table 8:** Initial tuning parameters of rain optimization algorithm (ROA).

Parameter	nPop	MaxIt	nVar	VarMin	VarMax	InitR	Speed	α
Value	100	100	1000	0	12,000	600	0	0

**Figure 27:**
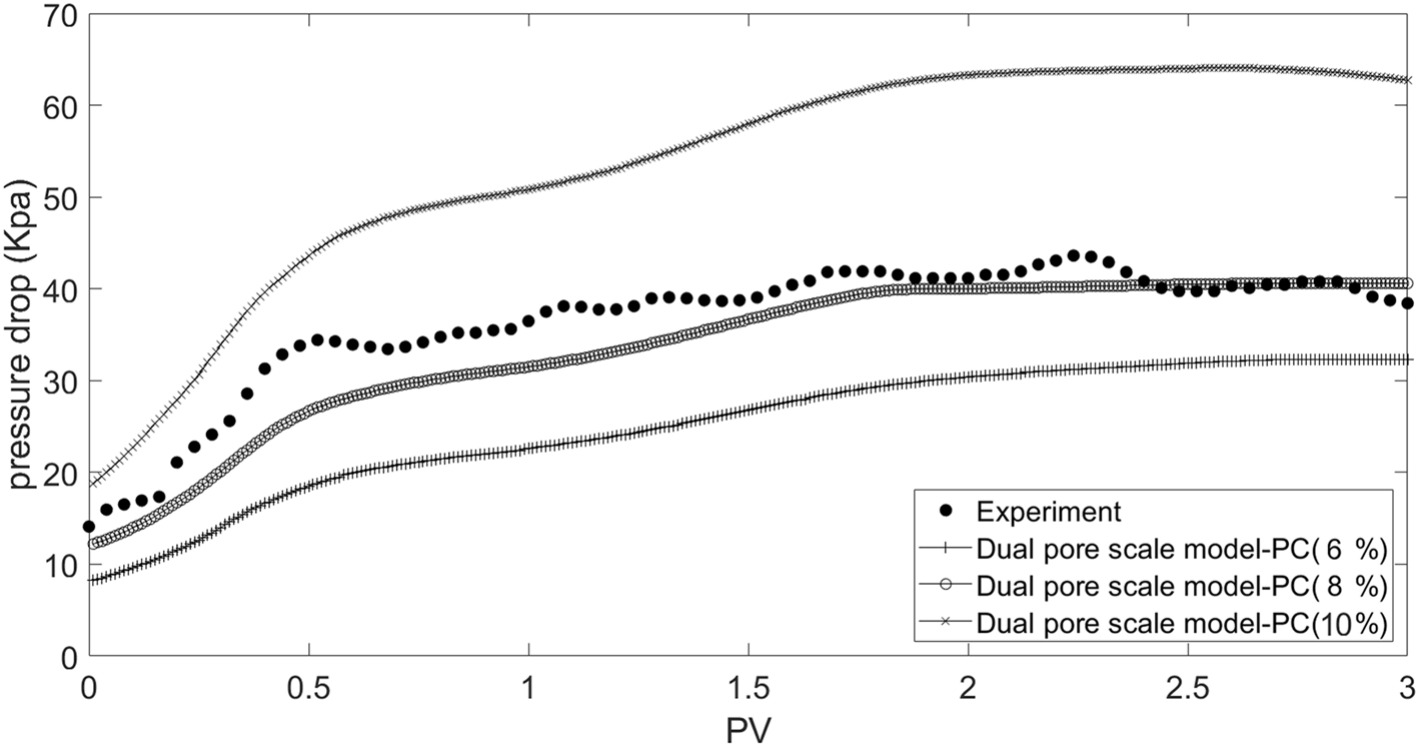
Matching dual pore scale model results and experimental data.

**Figure 28:**
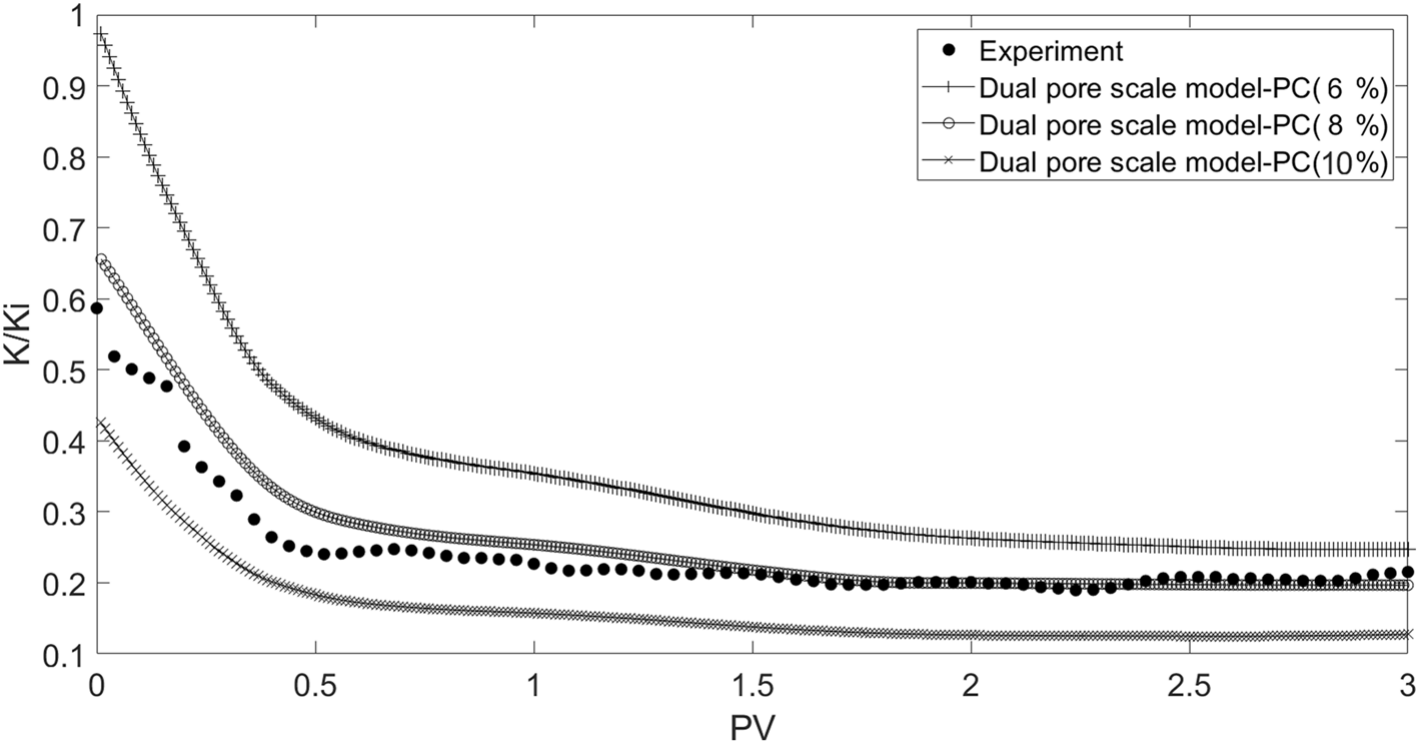
Matching dual pore scale model results and experimental data.

The modeling results for pressure drop and permeability damage as a function of injected pore volume are shown in Figs. 27 and 28, together with data from the core flooding experiment. According to the results, the best match is found in particles with a concentration of 8%, which is in line with the digital core analysis result of 8.36%.

## Conclusion

Core flood experiments have proven to be an efficient tool for research in reservoir engineering, if the results of these tests are accurate and have high quality. However, achieving core flooding results with high quality in laboratory conditions is not easy due to uncertainties such as instrumental and manual error. Also, implementation of the core flooding experiment in reservoir conditions is a time-consuming approach. Therefore, providing a rapid alternative approach for core flood experiment with suitable accuracy and lower cost seems necessary.

In this work, a rapid, accurate, and low-cost approach was developed to modeling the particle-fluid flow in the core sample (porous media) for water flooding process optimization in an Iranian oil reservoir. This approach is demonstrated to be an appropriate replacement for core flooding experiments when the results of this modeling approach are compared with the data obtained from the experiments.

Our key conclusions are listed below in brief:

- Prior to implementing water flooding in the field, it is crucial to optimize the salinity and ionic composition of the injected seawater since these factors have a significant impact on the permeability damage. Additionally, the composition of the injected saltwater contains more Mg^2+^ and less SO_4_^2−^, which prevents the growth of additional scales and reduces permeability damage.
- The parameter of contact time between formation water and injected water has a strong impact on the permeability damage severity, so the effect of this parameter should be considered for accurate evaluation of permeability damage in the core flood experiment.
- In our case study, determination accuracy of porous media structure parameters using 2-D binary image and SEM image analysis is high enough and maximum error related to experimental measurement is about 5%.
- Computational efficiency of our model by applying the proxy model approach and rain optimization algorithm as well as accuracy of our model by adding the various particles capture mechanisms to the DPM model is high enough, so this approach is a suitable choice for formation damage studies, especially when there are limitations on time and facilities.

## Data Availability

The datasets used and/or analysed during the current study available from the corresponding author on reasonable request.

## Abbreviations

CFD: Computational fluid dynamic
$${V}_{c}$$: Critical velocity, m/s
$$\mathrm{D}$$: Microchannel diameter, m
${\uptau }_{w}$: Wall shear stress, pa
$\mu$: Dynamic viscosity, Pa.s
${\rho }_{p}$: Particle density, kg/m^3^
$\rho$: Fluid density, kg/m^3^
$${d}_{p}$$: Particle diameter, m
*b*: Particle deformation normal to surface, m
$a$: Particle deformation along the surface, m
$$F$$: Body force, N
${u}^{*}$: Wall shear velocity, m/s
$$V$$: Fluid velocity, m/s
$$P$$: Pressure, Pa
є: Relative dielectric constant of water
${\upepsilon }_{0}$: Dielectric constant in vacuum
δ: Separation distance between the particle and surface, m
${\upzeta }_{p}$: Zeta potentials of the particle, mV
${\upzeta }_{\mathsf{g}}$: Zeta potentials of the surface, mV
$\kappa$: Debye reciprocal double layer thickness, m
$I$: Ionic concentration in the water, mol/L
${N}_{A}$: Avogadro’s number, 1/mols
$$e$$: Elementary charge, C
$$T$$: Absolute temperature, K
$$\mathtt{k}$$: Boltzmann’s constant
${W}_{A}$: Work of adhesion, J/m^2^
${K}_{c}$: Composite young’s modulus, 1/pa
${\vartheta }_{s}$: Poisson’s ratio value of the wall surface
${\vartheta }_{p}$: Poisson’s ratio value of the particle
${E}_{s}$: Young’s modulus value of the wall surface, pa
${E}_{p}$: Young’s modulus value of the particle, pa
$$H$$: Hamaker constant
${H}_{p}$: Hamaker constant of the particle
${H}_{w}$: Hamaker constant of the water
${H}_{s}$: Hamaker constant of the sand
${K}_{s}$: Static friction coefficient
$${P}_{r}$$: Collision probability
$$r$$: Particles radius, m
${v}_{rel}$: Particles relative velocity, m/s
$${\Delta }_{t}$$: Time step
${V}_{cp}$: Volume of the continuous-phase cell, m^3^
${b}_{act}$: Actual collision parameter
$$Y$$: Random number
$${b}_{crit}$$: Critical offset
$f$: Relative radius function
${W}_{e}$: Collisional Weber number
${T}_{d}$: Average thickness of deposited layer, m
$Ap$: Deposited particle area, m^2^
$$L$$: Microchannel length, m
PC: Particles concentration
P/D: Ratio of particle mean diameter to microchannel diameter
L/D: Ratio of microchannel length to microchannel diameter
Re: Reynolds number
ANN: Artificial neural networks
${g}_{ij}$: Hydraulic conductivity of the throat, m/s
ROA: Rain optimization algorithm
nPop: Population number
MaxIt: Maximum iteration
nVar: Number of variables
VarMin: Lower bound of variables
VarMax: Upper bound of variables
InitR: Initial radius of droplet
α: Soil adsorption constant
CT: Contact time

## References

[1] Kazemi, N. K. A. in *Mechanistic Modeling of Low Salinity Water Injection* (2014).

[2] Shalabi EWA, Sepehrnoori K, Delshad M (2014). Mechanisms behind low salinity water injection in carbonate reservoirs. Fuel.

[3] Yousef, A. A., Al-Saleh, S., Al-Kaabi, A. & Al-Jawfi, M. Laboratory investigation of novel oil recovery method for carbonate reservoirs. In *Canadian Unconventional Resources and International Petroleum Conference* p. SPE-137634-MS. 10.2118/-MS (2010)

[4] Laing, N., Graham, G. M. & Dyer, S. J. Barium sulphate inhibition in subsea systems—The impact of cold seabed temperatures on the performance of generically different scale inhibitor species. In *International Symposium on Oilfield Chemistry* p. SPE-80229-MS. 10.2118/-MS (2003).

[5] Sorbie, K. S. & Laing, N. How scale inhibitors work: Mechanisms of selected barium sulphate scale inhibitors across a wide temperature range. In *SPE International Symposium on Oilfield Scale* p. SPE-87470-MS. 10.2118/-MS (2004).

[6] Khaksar Manshad A, Olad M, Taghipour SA, Nowrouzi I, Mohammadi AH (2016). Effects of water soluble ions on interfacial tension (IFT) between oil and brine in smart and carbonated smart water injection process in oil reservoirs. J. Mol. Liq..

[7] Manshad AK, Nowrouzi I, Mohammadi AH (2017). Effects of water soluble ions on wettability alteration and contact angle in smart and carbonated smart water injection process in oil reservoirs. J. Mol. Liq..

[8] Moghadasi, J., Jamialahmadi, M., Müller-Steinhagen, H. & Sharif, A. Scale formation in oil reservoir and production equipment during water injection kinetics of CaSO_4_ and CaCO_3_ crystal growth and effect on formation damage. In: SPE European Formation Damage Conference. p. SPE-82233-MS. 10.2118/-MS (2003).

[9] Tahmasebi HA, Kharrat R, Soltanieh M (2010). Dimensionless correlation for the prediction of permeability reduction rate due to calcium sulphate scale deposition in carbonate grain packed column. J. Taiwan Inst. Chem. Eng..

[10] Yassin, A. A. M., Ali, N. M. & Merdhah, A. B. B. (Eds). *Formation Damage Due to Scale Formation in Porous Media Resulting from Water Injection* (2008).

[11] Mohammadi M, Riahi S (2020). Experimental investigation of water incompatibility and rock/fluid and fluid/fluid interactions in the absence and presence of scale inhibitors. SPE J..

[12] Kan AT, Tomson MB (2012). Scale prediction for oil and gas production. SPE J..

[13] Mackay, E. J. & Graham, G. M. The use of flow models in assessing the risk of scale damage. In *International Symposium on Oilfield Chemistry* p. SPE-80252-MS. 10.2118/-MS (2003)

[14] Talaei, A. & Moghadasi, J. Experimental investigation of scale formation and prediction by a novel method, electrical conductivity. In *First International Conference of Oil, Gas, Petrochemical and Power Plant*; Tehran (2012).

[15] Naseri S, Moghadasi J, Jamialahmadi M (2015). Effect of temperature and calcium ion concentration on permeability reduction due to composite barium and calcium sulfate precipitation in porous media. J. Nat. Gas Eng..

[16] Riepe, L., Suhaimi, M. H., Malaysia, M. K. & Knackstedt, M. A. Application of high resolution micro-CT imaging and pore network modeling (PNM) for the petrophysical characterization of tight gas reservoirs—A case history from a deep clastic tight gas reservoir in Oman. In *SPE Middle East Unconventional Gas Conference and Exhibition* p. SPE-142472-MS. 10.2118/-MS (2011).

[17] Mohd Zainudin, W. N. S. B. W., Zain, Z. M. & Riepe, L. Application of digital core analysis (DCA) and pore network modeling (PNM) based on 3D micro-CT images for an EOR project in a mature oil field in East Malaysia. In *International Petroleum Technology Conference* p. IPTC-17585-MS. 10.2523/IPTC--MS (2014)

[18] Hasnan HK, Sheppard A, Hassan MHA, Abdullah WH (2020). Digital core analysis: Characterizing reservoir quality through thin sandstone layers in heterolithic rocks. Appl. Geophys..

[19] Rabbani A, Jamshidi S, Salehi S (2014). An automated simple algorithm for realistic pore network extraction from micro-tomography images. J. Pet Sci. Eng..

[20] Rabbani A, Ayatollahi S, Kharrat R, Dashti N (2016). Estimation of 3-D pore network coordination number of rocks from watershed segmentation of a single 2-D image. Adv. Water Resour..

[21] Rabbani A, Assadi A, Kharrat R, Dashti N, Ayatollahi S (2017). Estimation of carbonates permeability using pore network parameters extracted from thin section images and comparison with experimental data. J. Nat. Gas Eng..

[22] Rabbani A, Salehi S (2017). Dynamic modeling of the formation damage and mud cake deposition using filtration theories coupled with SEM image processing. J. Pet Sci. Eng..

[23] Ezeakacha, C. P., Rabbani, A., Salehi, S., Ghalambor, A. Integrated image processing and computational techniques to characterize formation damage. In: SPE International Conference and Exhibition on Formation Damage Control p. D012S07R04. 10.2118/189509-MS (2018)

[24] Bagrezaie MA, Dabir B, Rashidi F (2022). A novel approach for pore-scale study of fines migration mechanism in porous media. J. Pet. Sci. Eng..

[25] Soltani M, Ahmadi G (1994). On particle adhesion and removal mechanisms in turbulent flows. J. Adhes. Sci. Technol..

[26] Podczeck F (1998). Particle-Particle Adhesion in Pharmaceutical Powder Handling.

[27] Van Oss CJ, Good RJ, Chaudhury MK (1986). The role of van der Waals forces and hydrogen bonds in “hydrophobic interactions” between biopolymers and low energy surfaces. J. Colloid Interface Sci..

[28] Derjaguin BV, Aleinikova IN, Toporov YP (1994). On the role of electrostatic forces in the adhesion of polymer particles to solid surfaces. Prog. Surf. Sci..

[29] Bai R, Tien C (1997). Particle detachment in deep bed filtration. J. Colloid Interface Sci..

[30] Tien C, Ramarao BV (2011). Granular Filtration of Aerosols and Hydrosols.

[31] Chen Y, Ma J, Wu X, Weng L, Li Y (2020). Sedimentation and transport of different soil colloids: Effects of goethite and humic acid. Water.

[32] Henry C, Minier J-P, Pozorski J, Lefèvre G (2013). A new stochastic approach for the simulation of agglomeration between colloidal particles. Langmuir.

[33] Zhang J, Mi J, Wang H (2012). A new mesh-independent model for droplet/particle collision. Aerosol Sci. Technol..

[34] Khilar, K. C. & Fogler, H. S. (Eds). in *Migrations of Fines in Porous Media* (1998).

[35] Jung J, Cao SC, Shin Y-H, Al-Raoush RI, Alshibli K, Choi J-W (2018). A microfluidic pore model to study the migration of fine particles in single-phase and multi-phase flows in porous media. Microsyst. Technol..

[36] Damean N, Regtien PPL (2001). Poiseuille number for the fully developed laminar flow through hexagonal ducts etched in 〈1 0 0〉 silicon. Sens Actuator A Phys..

[37] ANSYS. Academic Research release 18.0 Help system 24.6.12 Coupled Calculations. ANSYS,INC (2016)

[38] Carreras, P. E., Turner, S. E. & Wilkinson, G. T. Tahiti: Development strategy assessment using design of experiments and response surface methods. In *SPE Western Regional/AAPG Pacific Section/GSA Cordilleran Section Joint Meeting* p. SPE-100656-MS. 10.2118/-MS (2006)

[39] Slotte, P. A. & Smørgrav, E. Response surface methodology approach for history matching and uncertainty assessment of reservoir simulation models. In *Europec/EAGE Conference and Exhibition*. p. SPE-113390-MS. 10.2118/-MS (2008)

[40] Narayanan, K., White, C. D., Lake, L. W. & Willis, B. J. Response surface methods for upscaling heterogeneous geologic models. In *SPE Reservoir Simulation Symposium* p. SPE-51923-MS. 10.2118/-MS (1999)

[41] Shams, M., El-Banbi, A. H. & Sayyouh, H. A comparative study of proxy modeling techniques in assisted history matching. In *SPE Kingdom of Saudi Arabia Annual Technical Symposium and Exhibition* p. D033S20R03. 10.2118/188056-MS (2017)

[42] Cranganu C, Luchian H, Breaban ME (2015). Artificial Intelligent Approaches in Petroleum Geosciences.

[43] Fatt IJTotA. The Network Model of Porous Media. **207**, 144–181 (1956).

[44] Balhoff MT, Thompson KE, Hjortsø M (2007). Coupling pore-scale networks to continuum-scale models of porous media. Comput. Geosci..

[45] Thompson KE, Fogler HS (1997). Modeling flow in disordered packed beds from pore-scale fluid mechanics. AIChE J..

[46] Moazzeni AR, Khamehchi E (2020). Rain optimization algorithm (ROA): A new metaheuristic method for drilling optimization solutions. J. Pet Sci. Eng..

